# *Cdkn1c* Boosts the Development of Brown Adipose Tissue in a Murine Model of Silver Russell Syndrome

**DOI:** 10.1371/journal.pgen.1005916

**Published:** 2016-03-10

**Authors:** Matthew Van De Pette, Simon J. Tunster, Grainne I. McNamara, Tatyana Shelkovnikova, Steven Millership, Lindsay Benson, Stuart Peirson, Mark Christian, Antonio Vidal-Puig, Rosalind M. John

**Affiliations:** 1 School of Biosciences, Cardiff University, Cardiff, United Kingdom; 2 MRC Clinical Sciences Centre, Hammersmith Hospital, London, United Kingdom; 3 Nuffield Department of Clinical Neuroscience, Nuffield Laboratory of Ophthalmology, John Radcliffe Hospital, Oxford, United Kingdom; 4 Division of Translational and Systems Medicine, Warwick Medical School, University of Warwick, Coventry, United Kingdom; 5 Metabolic Research Laboratories, Institute of Metabolic Science, Addenbrooke’s Hospital, Cambridge, United Kingdom; University of Pennsylvania, UNITED STATES

## Abstract

The accurate diagnosis and clinical management of the growth restriction disorder Silver Russell Syndrome (SRS) has confounded researchers and clinicians for many years due to the myriad of genetic and epigenetic alterations reported in these patients and the lack of suitable animal models to test the contribution of specific gene alterations. Some genetic alterations suggest a role for increased dosage of the imprinted *CYCLIN DEPENDENT KINASE INHIBITOR 1C* (*CDKN1C*) gene, often mutated in IMAGe Syndrome and Beckwith-Wiedemann Syndrome (BWS). *Cdkn1c* encodes a potent negative regulator of fetal growth that also regulates placental development, consistent with a proposed role for *CDKN1C* in these complex childhood growth disorders. Here, we report that a mouse modelling the rare microduplications present in some SRS patients exhibited phenotypes including low birth weight with relative head sparing, neonatal hypoglycemia, absence of catch-up growth and significantly reduced adiposity as adults, all defining features of SRS. Further investigation revealed the presence of substantially more brown adipose tissue in very young mice, of both the classical or canonical type exemplified by interscapular-type brown fat depot in mice (iBAT) and a second type of non-classic BAT that develops postnatally within white adipose tissue (WAT), genetically attributable to a double dose of *Cdkn1c in vivo* and *ex-vivo*. Conversely, loss-of-function of *Cdkn1c* resulted in the complete developmental failure of the brown adipocyte lineage with a loss of markers of both brown adipose fate and function. We further show that *Cdkn1c* is required for post-transcriptional accumulation of the brown fat determinant PR domain containing 16 (PRDM16) and that CDKN1C and PRDM16 co-localise to the nucleus of rare label-retaining cell within iBAT. This study reveals a key requirement for *Cdkn1c* in the early development of the brown adipose lineages. Importantly, active BAT consumes high amounts of energy to generate body heat, providing a valid explanation for the persistence of thinness in our model and supporting a major role for elevated *CDKN1C* in SRS.

## Author Summary

Silver Russell syndrome is a severe developmental disorder characterised by low birth weight, sparing of the head and neonatal hypoglycemia. SRS adults are small and can be extremely thin, lacking body fat. Numerous genetic and epigenetic mutations have been linked to SRS primarily involving imprinted genes, but progress has been hampered by the lack of a suitable animal model. Here we describe a mouse model of the rare micro duplications reported in some SRS patients, which recapitulated many of the defining features of SRS, including extreme thinness. We showed that these mice possessed substantially more of the energy consuming brown adipose tissue (BAT), driven by a double dose of the imprinted *Cdkn1c* gene. We further show that Cdkn1c is required for the postranscriptional accumulation of the BAT determinant PRDM16 and that these proteins co-localise to the nucleus of in a rare label-retaining cell within BAT. These data suggest that Cdkn1c contributes to the development of BAT by modulating PRDM16 and supports a major role for this gene in SRS.

## Introduction

Silver-Russell syndrome (SRS; MIM 180860), Beckwith Weidemann Syndrome (BWS; MIM 130650) and IMAGe syndrome (MIM 614732) are all rare imprinted developmental disorders that occur as a result of genetic or epigenetic alterations primarily at human chromosome 11p15 [1, 2]. Recent studies highlight the potential involvement of one maternally expressed imprinted gene, *CYCLIN DEPENDENT KINASE INHIBITOR 1C* (*CDKN1C)*, in all three disorders [3]. Loss-of-function or loss-of-expression of *CDKN1C* is a common feature of BWS, either through direct DNA mutation, epigenetic misregulation or loss of the maternal chromosome [4]. The rare IMAGe syndrome, which has the major features of fetal growth restriction, metaphyseal displasia, adrenal hypoplasia congentia and genital abnormalities, is associated with genetic mutations in the *CDKN1C* gene [5, 6]. The changes associated with growth restriction are gain-of-function mutations of the PCNA domain, limited to a handful of rare familial cases highlighted in a recent review [3], that may increase the stability of the protein [6, 7]. SRS is characterised by severe pre and post natal growth restriction combined with some of the following: neonatal hypoglycaemia, excessive sweating, triangular shaped face, head circumference of normal size but disproportionate to a small body size, clinodactyly, feeding problems, low body mass index manifesting as extreme thinness, no catch up growth and increased risk of delayed development and learning disabilities [8]. Numerous genetic and epigenetic alterations have been reported in SRS patients but identifying the causal gene mutation(s) has been challenging. Some studies suggest loss of function of the paternally expressed growth factor *INSULIN-LIKE GROWTH FACTOR 2* (*IGF2*) [9]. However, there are SRS patients that carry an extra copy of maternally derived 11p15 without loss-of-function of *IGF2* [10]. Maternal duplications spanning the complex imprinted domain at 11p15 have been independently reported in a number of studies [11–16] and the majority are associated with unbalanced translocations suggesting that increased dosage of a maternally expressed imprinted gene may be important in SRS. The minimal region of maternal microduplication in SRS encompasses *CDKN1C* and three other maternally expressed protein coding genes *POTASSIUM CHANNEL*, *VOLTAGE GATED KQT-LIKE SUBFAMILY Q*, *MEMBER 1* (*KCNQ1)*, *PLECKSTRIN HOMOLOGY-LIKE DOMAIN*, *FAMILY A*, *MEMBER 2* (*PHLDA2)* and *SOLUTE CARRIER FAMILY 22*, *MEMBER 18* (*SLC22A18)* [17, 18]. Since *CDKN1C* is a maternally expressed gene [19, 20], these SRS patients are predicted to have twice the normal level of *CDKN1C* expression.

We, and others, have shown that loss of *Cdkn1c* in mice results in a late fetal overgrowth and disrupted placental development [21–25] consistent with a key role for this gene in BWS. *CDKN1C*, which is maternally expressed in both humans and mice [19, 20], belongs to the Kip cyclin dependent kinase inhibitor family that induce cell cycle arrest and limit proliferation [26]. In mice, *Cdkn1c* is widely expressed during embryonic development in cells exiting differentiation [27–29]. *Cdkn1c* also functions to orchestrate cell fate determination targeting key transcription factors [30–36] and in stem cell self-renewal and quiescence in a number of embryonic [32–34, 37–40] and adult [41–43] stem/progenitor cells. These multiple roles may account for complex phenotypic consequences in response to alterations in the dosage of this gene.

We previously reported growth restriction in mice carrying a bacterial artificial chromosome (BAC) transgene spanning *Cdkn1c*, *Phlda2* and *Slc22a18* [24]. This alteration essentially models the minimal microduplicated region observed in some SRS patients [17, 18]. The mice exhibited significant fetal growth restriction from embryonic day (E) 13.5 with the absence of catch-up growth. We were able to attribute the fetal growth restricting properties of this microduplication to two-fold expression of *Cdkn1c* consistent with the phenotype observed in SRS. Fetal growth restriction *per se* is a relatively generic phenotype and more specific features of SRS would lend greater support to the hypothesis that altered expression of *CDKN1C* contributes significantly to SRS in human patients. To provide further evidence for or against a key role for *CDKN1C* in SRS, we examined the microduplication mice for additional SRS-associated phenotypes. This work revealed low birth weight with a relative sparing of the head, neonatal hypoglycaemia, small sized adults with substantially less white adipose tissue, all of which were genetically attributable to just two-fold expression of *Cdkn1c*. These findings support a major role for elevated *CDKN1C* in SRS. Importantly, we identified a novel function for *Cdkn1c* in directly promoting the development of brown adipose tissue early in life, a finding that could account for the prevalence of thinness in SRS.

## Results

**Fig 1:**
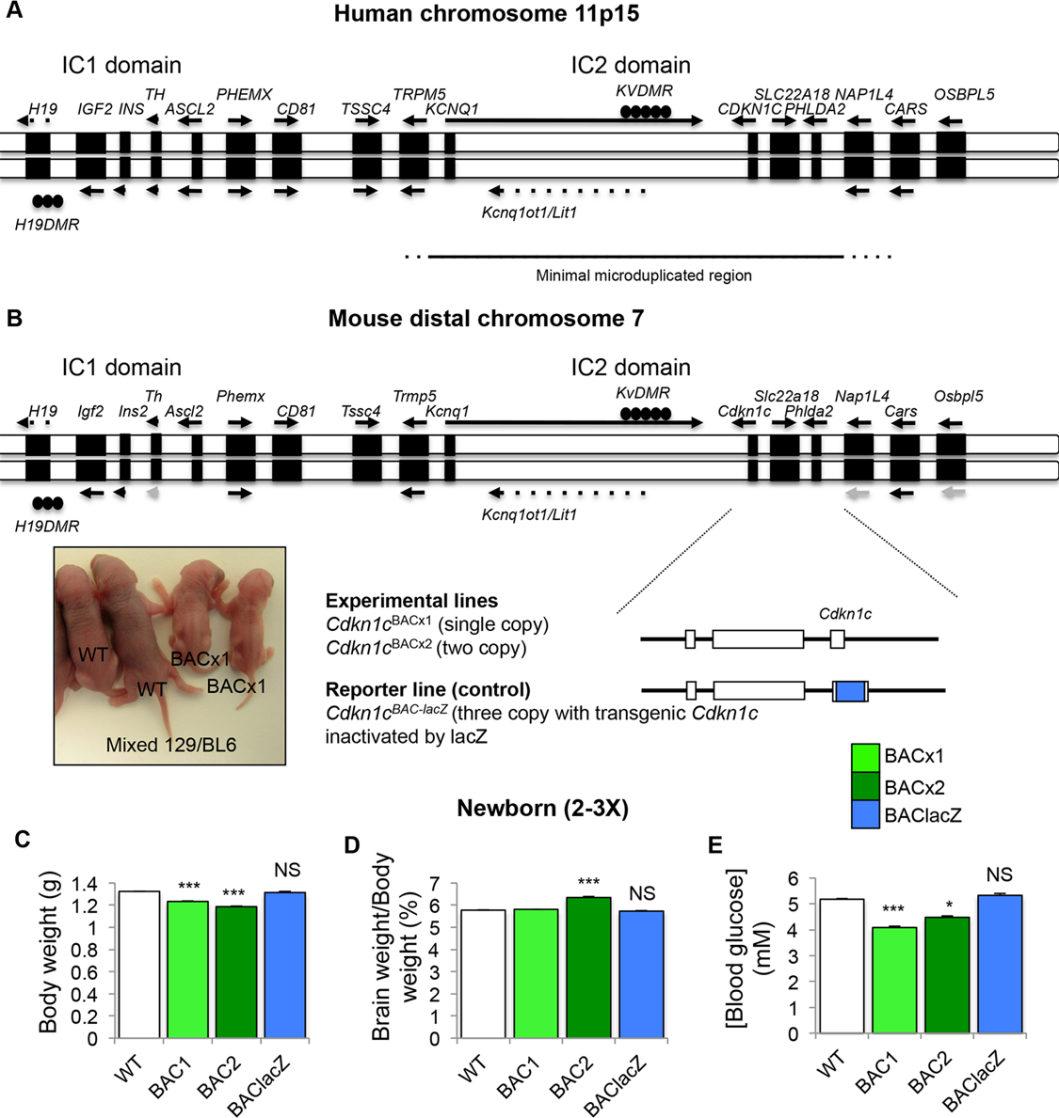
Bacterial artificial chromosome (BAC) spanning the intact *Cdkn1c* locus in mice models minimal microduplication reported in Silver Russell Syndrome. (A) Genomic map of human 11p15 imprinted region. Line below indicates extent of minimal region duplicated in SRS. (B) Genomic map of mouse distal chromosome 7 imprinted region. Below is the map of the 85 kb BAC transgene (BAC144D14). Inset: Image of WT and BACx1 pups carrying one copy of the BACx1 examined on a mixed 129/BL6 genetic background on postnatal day (P) 2. (C) Weights of WT and BAC transgenic pups at birth (P0) after breeding onto BL6 genetic background for >12 generations. (D) Brain weight to body weight ratio. (E) Blood glucose levels (mmol/l). NS = not significant. Data expressed as mean ± SEM, *t* test. Numbers given in **S1 Fig**.

The minimal microduplicated region in SRS spans four imprinted, protein-coding genes: *KCNQ1*, *CDKN1C*, *PHLDA2* and *SLC22A18* (**Fig 1A**). Our mouse BAC transgene spans three of these genes, *Cdkn1c*, *Phlda2* and *Slc22a18* (**Fig 1B**). Previously we showed that a single copy of the transgene (BACx1) drove significant fetal growth restriction on a mixed strain background which was more severe in a two copy line (BACx2) and absent in a reporter line in which transgenic *Cdkn1c* was replaced by a *β-galactosidase* gene (BAC-lacZ) attributing growth restriction to elevated expression of *Cdkn1c* [24]. The transgene was lethal on a pure 129S2/SvHsd (129) background but preliminary breeding into C57BL/6J (BL6) suggested improved viability with the retention of growth restriction, albeit attenuated [24]. For this study, three transgenic lines (BACx1, BACx2 and BAC-lacZ) were bred further onto a BL6 strain background, to >12 generations. BACx1 and BACx2 fetuses were significantly lighter at embryonic day (E) 18.5 while BAC-lacZ fetuses were similar in weight to wild type controls confirming the fetal growth restricting properties of *Cdkn1c*. Importantly, fetal viability was not compromised on this genetic background (**S1 Fig**).

Children with SRS are born low birth weight and are prone to develop spontaneous hypoglycaemia, particularly if they are not fed both frequently and regularly [44]. Newborn BACx1 and BACx2 mice were lighter than their wild type littermates (**Fig 1C**). There was a significant difference in the relative proportion of the brain to body weight in the two copy line (**Fig 1D**). Marked hypoglycaemia in the fed state was evident for both the single copy and the two copy line (**Fig 1E**). Glucose levels, birth weight and brain weights were normal in the control line BAC-lacZ genetically attributing these phenotypes to elevated *Cdkn1c* expression. These data were consistent with the observed phenotypes of low birth weight, head sparing and neonatal hypoglycaemia reported in young SRS patients.

**Fig 2:**
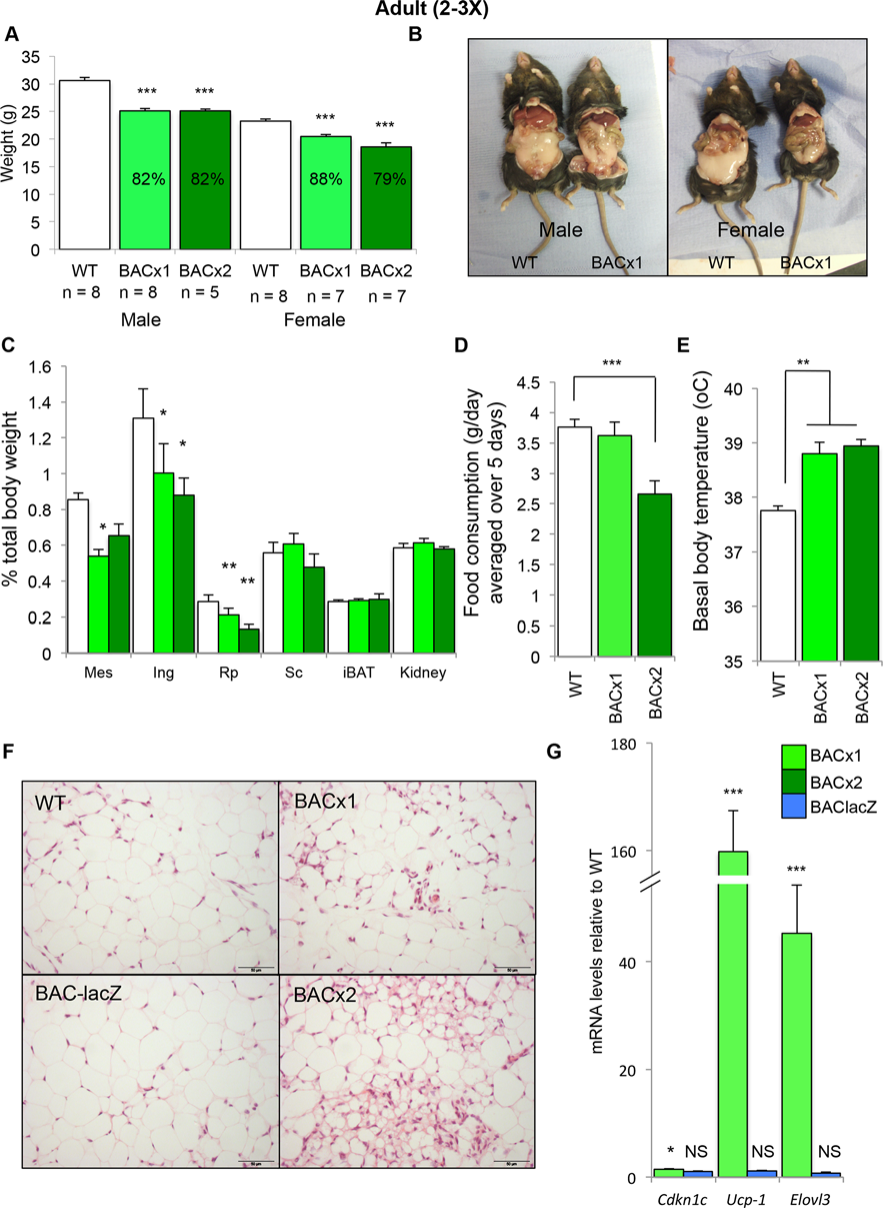
Elevated *Cdkn1c* drives thinness in adult mice. (A) Weights of WT and BAC transgenic male and female mice at 10 weeks. (B) Dissection of WT and BAC transgenic mice at 10 weeks to reveal adipose depots *in situ*. (C) Weights of adipose depots relative to body weights. (D) Food consumption per day, measurements taken over 5 days. (E) Rectal body temperature. (F) H&E sections of 10 week rpWAT depots from WT, BACx1 and BACx2 and BAClacZ (WT from line BACx1). (G) QPCR analysis of *Cdkn1c*, *Ucp1* and *Elovl3* in BACx1 female 10 week rpWAT depots (n = 4 per genotype). Data expressed as mean ± SEM, *t* test.

As adults, SRS patients commonly display short stature with a low body mass index and a lack of subcutaneous fat [8]. At 10 weeks of age BACx1 and BACx2 male and female adult mice were significantly lighter than their wild type littermates (**Fig 2A**). An exploratory magnetic resonance image of an adult BACx1 male mouse alongside a wild type co-housed littermate suggested less white adipose tissue (WAT; **S2 Fig**). Dissection and weighing of individual WAT depots revealed a disproportionate reduction in the weight of the mesenteric (mes), inguinal (ing) and retroperitoneal (rp) WAT depots relative to total body weight (**Fig 2B and 2C**). Mice carrying a single copy of the transgene (BACx1) consumed a similar daily weight of standard chow whereas mice carrying two copies (BACx2) consumed significantly less (**Fig 2D**). The basal body temperature of both BACx1 and BACx2 mice was significantly elevated (**Fig 2E**). Histological examination of rpWAT revealed an abundance of smaller adipocytes with a multilocular appearance, which were less apparent in wild type rpWAT and depots from the reporter line BAC-lacZ (**Fig 2F**). BEIGE cells are a recruitable form of brown adipose tissue (BAT) that develops postnatally within some WAT depots defined by a smaller cell size, multilocular lipid droplet morphology, a high mitochondrial content and the expression of brown fat–specific genes [45–50]. In addition to the elevated expression of *Cdkn1c* two key markers of brown adipose tissue, *uncoupling protein-1* (*UCP1*) and *elongation of very long chain fatty acids (FEN1/Elo2*, *SUR4/Elo3* and *yeast)-like 3 (Elovl3*), were significantly elevated in rpWAT from adult BACx1 mice as compared to matched wild type rpWAT (**Fig 2G**). Importantly, neither *Cdkn1c* nor these markers were elevated in rpWAT from the reporter line BAC-lacZ (**Fig 2G**). These data suggested an increased representation of BEIGE cells, sometimes referred to as the “browning” of WAT, driven by increased expression of *Cdkn1c*.

### *Cdkn1c* is expressed and imprinted in adipose tissue

**Fig 3:**
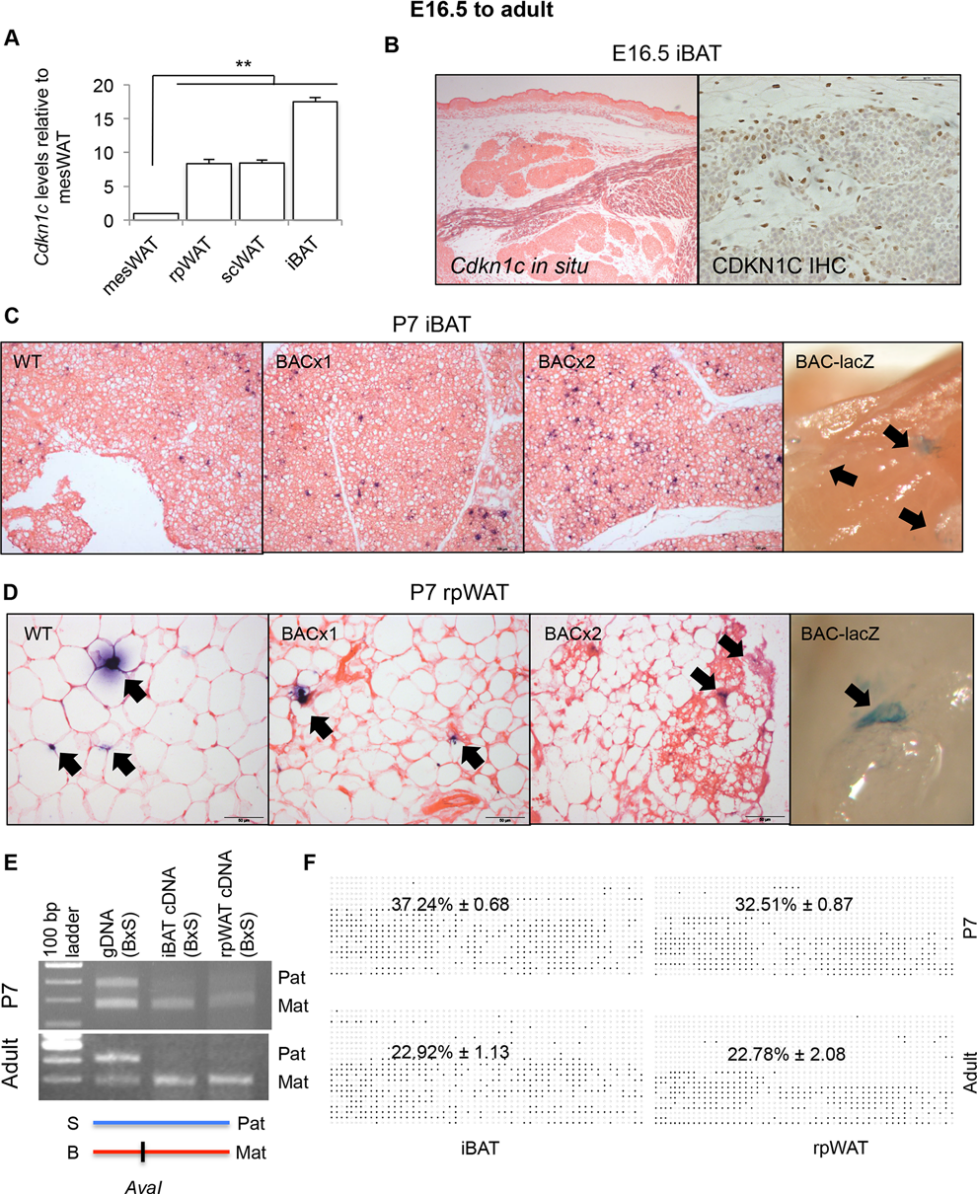
*Cdkn1c* is expressed and imprinted in rpWAT and iBAT. (A) QPCR of *Cdkn1c* in P7 rpWAT, subcutaneous (sc) WAT, and iBAT relative to mesenteric (mes) WAT (n = 4 each depot taken from two litters). Data expres sed as mean ± SEM, *t* test. ** *P* <0.01.(B) E16.5 transverse sections through IBAT depots stained for *Cdkn1c* mRNA and protein. (C) WT, BACx1 and BACx2 P7 iBAT sections stained for *Cdkn1c*. -galactosidase staining of P7 BAC-lacZ iBAT depot (far right panel). *Cdkn1c*-positive cells indicated by arrows. (D) WT, BACx1 and BACx2 P7 rpWAT sections stained for *Cdkn1c*. -galactosidase staining of P7 BAC-lacZ rpWAT depot (far right panel). *Cdkn1c*-positive cells indicated by arrows. (E) *Cdkn1c* maternal allele-specific expression in P7 and adult iBAT and rpWAT from hybrid offspring from BL6 female mated with a BL6^spretus-chr7^ male assessed by the presence (BL6; B) or absence (*spretus*; S) of an *AvaI* restriction enzyme site within the *Cdkn1c* PCR product. (F) Average methylated CpGs per sample with examples of differential methylation of *Cdkn1cDMR* in P7 and adult iBAT and rpWAT. Each row corresponds to an individual sequenced DNA clone. Each circle represents a CpG on the strand, filled circles and open circles indicate methylated and unmethylated sites, respectively. Percentage values given for n = 3 of each condition.

*Cdkn1c* is expressed from the BAC in a number of tissues including the pituitary, the hypothalamus and the pancreas [51] that may stimulate the browning of WAT. However, elevated *Cdkn1c* expression within transgenic rpWAT (**Fig 2G**) suggested the potential for a direct role for *Cdkn1c* in brown adipogenesis. Expression of *Cdkn1c* has been reported in the epididymal white adipose tissue of adult mice [52]. At postnatal day 7 (P7), *Cdkn1c* expression was detectable within several adipose depots with levels positively correlating with the brown adipose-like nature of these depots [53]. *Cdkn1c* was found to be most highly expressed in the interscapular-type brown fat depot (iBAT), which is composed of a classical or canonical type of BAT sharing a developmental origin with myoblasts [48], with moderate expression in rpWAT and subcutaneous (sc) WAT and lowest expression in mesenteric (mes) WAT (**Fig 3A**). At E16.5, when iBAT is discernable as a discrete depot, a few *Cdkn1c*-positive cells were identifiable by both *in situ* hybridisation and immunohistochemistry (**Fig 3B**). At P7, *Cdkn1c* was more widely expressed within the iBAT depot (**Fig 3C, left panel**). *Cdkn1c* was also expressed within a few discrete niches in P7 rpWAT (**Fig 3D, left panel**). Importantly, BACx1 and BACx2 rpWAT and iBAT displayed a similar expression pattern to WT depots by *in situ* hybridisation (**Fig 3C and 3D, middle panels**) indicating that *Cdkn1c* was not ectopically expressed from the transgene in these depots. *β-galactosidase* staining of dissected intact depots from BAC-lacZ pups revealed blue staining niches consistent with expression originating from the transgene in both depots (**Fig 3C and 3D, far right panels**).

To determine whether expression of *Cdkn1c* was imprinted in adipose tissue, we made use of the *Cdkn1c* restriction fragment length polymorphism (RFLP) assay [54]. *Mus musculus domesticus* BL6 mice possess an *AvaI* restriction enzyme site within an exon of *Cdkn1c* that is absent in *Mus spretus* mice (**Fig 3E**). P7 pups were generated from crosses between pure BL6 females and BL6 males carrying a copy of the *Mus spretus Cdkn1c* region. *AvaI* digestion of a PCR product amplified across the polymorphic region from genomic DNA demonstrated that both alleles were physically present. Digestion of the PCR product amplified from cDNA revealed the predominant presence of only the maternally inherited BL6 allele (lower band) in both P7 iBAT and P7 rpWAT (**Fig 3E**). Similarly, depots from adult mice displayed predominantly maternal-allele expression (**Fig 3E**). Differential DNA methylation spanning the predicted *Cdkn1c* promoter region [55] was also discernable in both adipose depots at P7 and in the adult (**Fig 3F**). These data demonstrated that *Cdkn1c* was both expressed and imprinted in post-natal adipose tissue, and that both expression and imprinting was maintained into adulthood.

### *Cdkn1c* promotes the browning of WAT early in life

**Fig 4:**
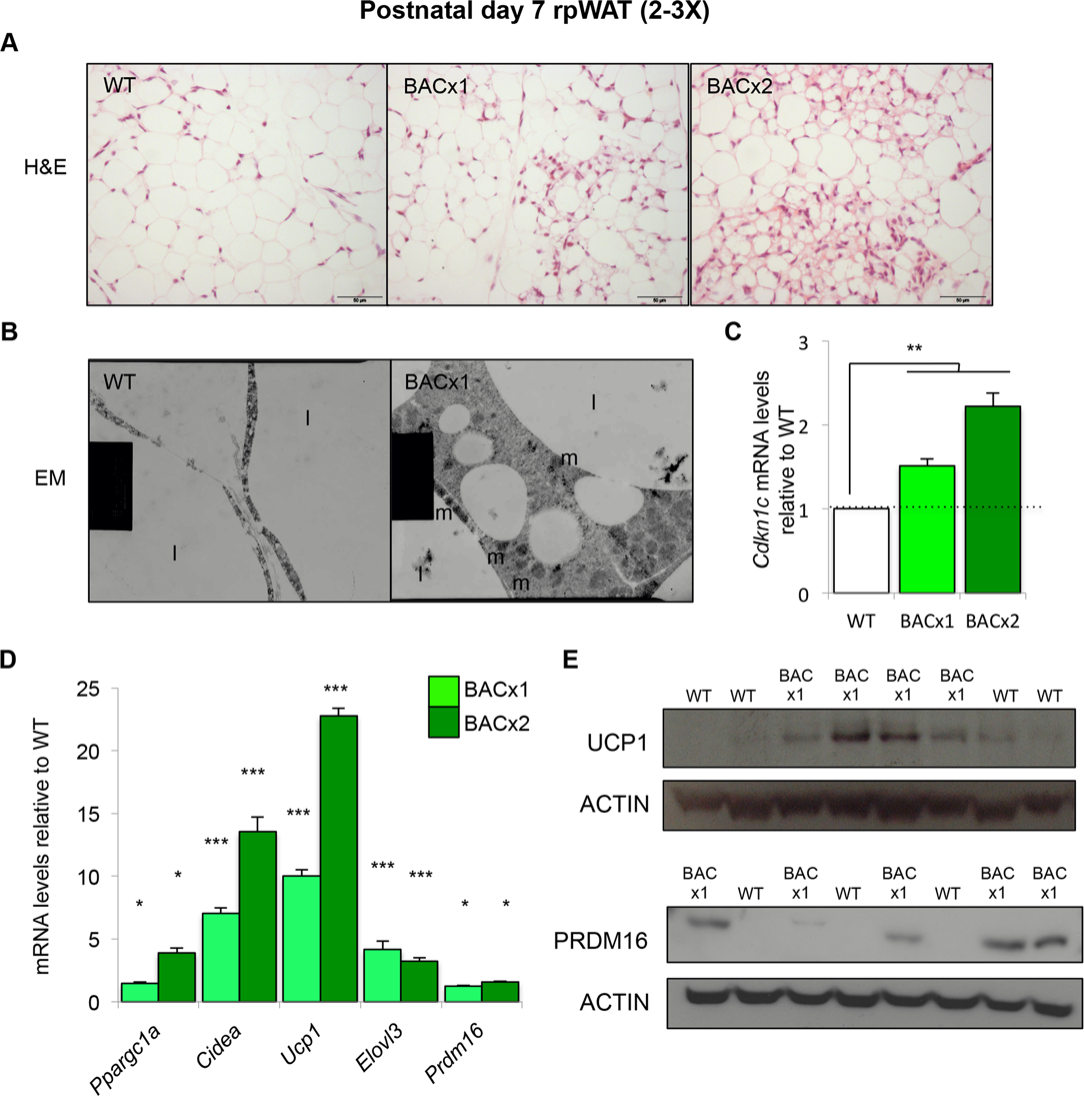
*Cdkn1c* promotes the browning of WAT in young mice. (A) H&E of P7 WT, BACx1 and BACx2 rpWAT (WT from line BACx1). (B) Electron micrograph of WT and BACx1 P7 rpWAT (4000X). Mitochondria indicated by m and lipid by l. (C) QPCR analysis of *Cdkn1c* mRNA levels in P7 rpWAT from BACx1 and BACx2 relative to wild type controls. (D) QPCR of BAT-selective genes in WT, BACx1 and BACx2 P7 rpWAT. (E) Western blot analysis of UCP1, PRDM16 and β-ACTIN in P7 rpWAT from single litters of WT and BACx1 pups. Data expressed as mean ± SEM, *t* test. * *P* <0.05; ** *P* <0.01; *** *P* <0.005.

The *in situ* hybridisation analysis (**Fig 3D**) and further histological examination of rpWAT at P7 suggested that the phenotypic differences present in adult mice were apparent at this much earlier timepoint (**Fig 4A**). Electron microscopic imaging of BACx1 P7 rpWAT depots revealed clusters of cells that possessed BEIGE characteristics including a larger volume of cytoplasm, numerous mitochondria and smaller, multilocular, lipid droplets not readily observed in matched WT depots (**Fig 4B**). QPCR demonstrated that *Cdkn1c* expression was significantly elevated in BACx1 and BACx2 P7 rpWAT, by 1.5- and 2.2-fold respectively (**Fig 4C**). Several markers of BAT were also elevated including *peroxisome proliferator-activated receptor gamma*, *coactivator 1 alpha* (*Ppargc1a*), *cell death-inducing DFFA-like effector a* (*Cidea*), *Ucp1*, *Elovl3* and *PR domain containing 16* (*Prdm16*) in BACx1 and BACx2 P7 rpWAT (**Fig 4D**). Consistent with 10-fold higher expression of *Ucp1* mRNA, UCP1 protein was more readily detectable in BACx1 rpWAT than in WT rpWAT in a within litter comparison (**Fig 4E**). PRDM16, a brown fat determinant [48], was also more readily detectable in BACx1 rpWAT depots than wild type depots (**Fig 4E**). Importantly, P7 rpWAT from the reporter line BAC-lacZ had a normal appearance and neither *Cdkn1c* nor key BAT markers were elevated (**S3A Fig**). The presence of BEIGE-like cells in the BACx1 and BACx2 rpWAT and their absence in the BAC-lacZ model, in which *Cdkn1c* was expressed at a normal level, identified *Cdkn1c* as a gene that promotes the “browning” of WAT. Importantly, this phenotype was apparent when WAT first emerged as a distinct depot in very young mice.

### *Cdkn1c* boosts the development of classic brown adipose tissue

**Fig 5:**
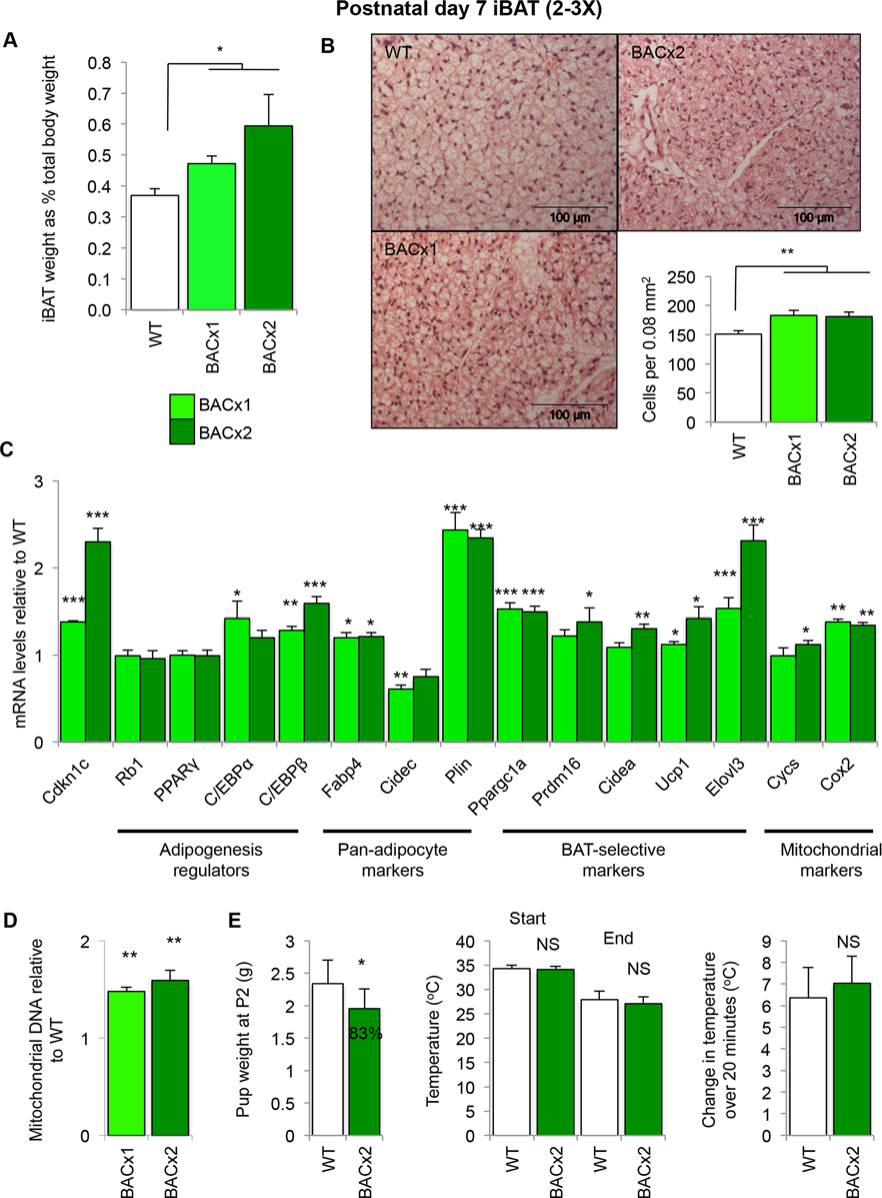
Elevated *Cdkn1c* boosts the formation of classic BAT in young mice. (A) Weights of WT, BACx1 and BACx2 iBAT relative to total body weight (WT n = 20, BACx1 n = 18, BACx2 n = 8). (B) H&E staining of P7 iBAT depot sections from WT, BACx1 and BACx2 pups (WT from line BACx1) and cell counting data (n = 6 per genotype). (C) QPCR of *Cdkn1c*, adipogenesis regulators, thermogenic, BAT-selective and mitochondrial genes in BACx1 and BACx2 P7 iBAT relative to WT (n = 4 per genotype). (D) Quantitation of mitochondrial genomic DNA of BACx1 and BACx2 iBAT relative to WT (n = 6). (E) Surface body temperature of BACx2 P2 pups relative to WT littermates was assessed by thermal imaging (WT n = 19, BACx2 n = 8) within 1 minute of removal from nest temperature (33°C; approaching thermoneutrality) and after 20 minutes at room temperature (22°C). Data expressed as mean ± SEM, *t* test. * *P* <0.05; ** *P* <0.01; *** *P* <0.005.

Elevated expression of *Cdkn1c* also had an effect on iBAT. P7 BACx1 and BACx2 iBAT depots were heavier as a proportion of total body weight, by 30% and 60% respectively, than WT iBAT depots (**Fig 5A**). This was not due to increased lipid deposition as BACx1 and BAC2 iBAT depots displayed increased cellularity, confirmed by cell counting (**Fig 5B**). As in rpWAT, *Cdkn1c* expression was significantly elevated in BACx1 and BACx2 iBAT, by 1.5- and 2.2-fold respectively (**Fig 5C**). QPCR analysis revealed near wild type expression of the adipogenesis regulators *retinoblastoma 1* (*Rb1*), *peroxisome proliferator-activated receptor-γ* (*PPARγ*) and *CCAAT-enhancer-binding protein-α* (*C/EBPα*) but elevated expression of *CCAAT-enhancer-binding protein-β* (*C/EBPβ)* in both BACx1 and BACx2 depots (**Fig 5C**). BAT markers *Ppargc1a*, *Ucp1* and *Elovl3* were significantly elevated in BACx1. All five BAT markers examined were significantly elevated in the higher dosage line, BACx2 (**Fig 5C**). Critically, BAC-lacZ iBAT appeared morphologically normal and neither *Cdkn1c* nor key BAT markers were elevated (**S3B Fig**) genetically assigning these alterations to the increased dosage of *Cdkn1c* in BACx1 and BACx2. The ratio of mitochondrial DNA to nuclear DNA can be used as an estimate of mitochondrial load. Both BACx1 and BAC2 P7 iBAT depots contained significantly increased mitochondrial DNA content compared to WT (**Fig 5D**). Consistent with a greater mitochondrial load, expression of the nuclear mitochondrial marker *cytochrome c*, *somatic* (*Cycs*) was significantly elevated in BACx2 and both *Cycs* and the mitochondrion-encoded *cytochrome c oxidase subunit II* (*Cox2*) were elevated in BAC1 and BACx2 iBAT (**Fig 5C**).

Fully functional iBAT at birth is important for maintaining newborn body temperature. 33°C approaches thermoneutrality and corresponds to the temperature within litters of newborn mice in contact with their mother [56] whereas 26°C elicits near-maximal thermogenesis by brown adipose tissue [57]. P2 WT and BACx2 pups kept at 33°C and then exposed to room temperature (22°C) for a 20 minute period both lost body heat at the same rate despite significant differences in their body weights (**Fig 5E**), consistent with functional iBAT at this timepoint.

Taken together, these data identified a novel function for *Cdkn1c* in boosting the development of both BEIGE and iBAT early in post-natal life, with increasing expression of *Cdkn1c* associated with the increased development of brown adipose.

### *Cdkn1c* is required for the proper formation of iBAT

**Fig 6:**
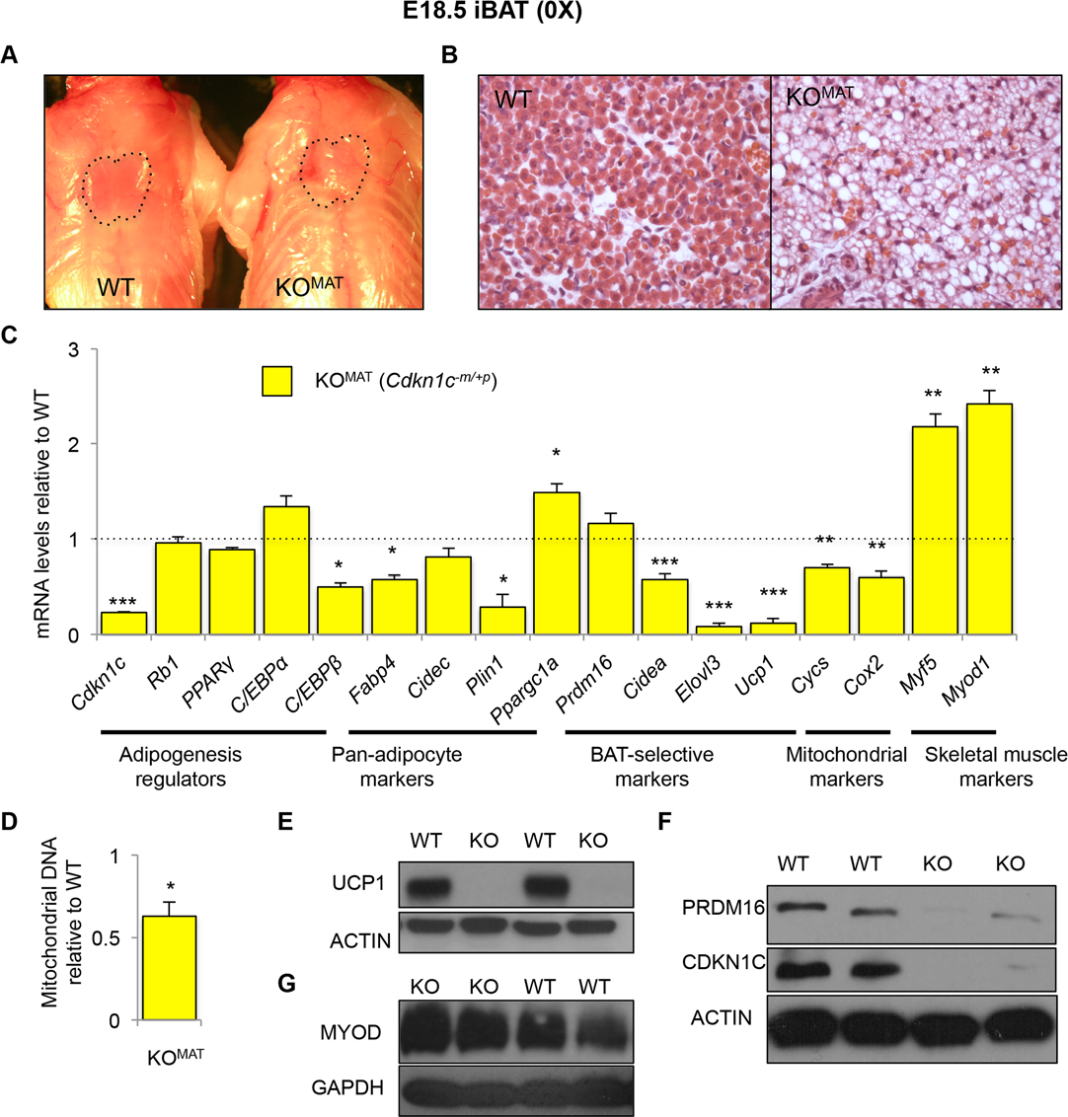
*Cdkn1c* is required for the proper formation of iBAT. (A) Photograph of E18.5 WT and *Cdkn1c*^*-/+*^ (KO^MAT^) fetuses with position of iBAT depot highlighted by dotted black line. (B) H&E staining of iBAT sections of E18.5 WT and KO^MAT^ iBAT. (C) QPCR of *Cdkn1c* and the adipocyte regulators *Rb1*, *PPARγ*, *C/EBPα* and *C/EBPβ*, pan-adipocyte genes *Fabp4*, *Cidec* and *Plin1*, BAT-selective genes *Ppargc1a*, *Cidea*, *Prdm16*, *Ucp1* and *Elovl3*, mitochondrial genes *Cycs* and *Cox2*, and the skeletal muscle-selective genes *Myf5* and *Myod1* in E18.5 KO^MAT^ iBAT relative to WT (n = 4 per genotype). (D) Mitochondrial DNA content of iBAT from E18.5 KO^MAT^ iBAT relative to WT (n = 6 per genotype). (E) Western blot analysis of UCP1 and β-ACTIN in E18.5 iBAT isolated from two WT and two KO^MAT^ fetuses. Within litter comparison. (F) Western blot analysis of CDKN1C, PRDM16 and β-ACTIN in E18.5 iBAT isolated from two WT and two KO^MAT^ fetuses. Within litter comparison. (G) Western blot analysis of MYOD and GAPDH in E18.5 E18.5 iBAT isolated from two WT and two KO^MAT^ fetuses. Within litter comparison. Data expressed as mean ± SEM, *t* test. * *P* <0.05; ** *P* <0.01; *** *P* <0.005.

In contrast to the increase in classic iBAT in response to elevated *Cdkn1c*, loss-of-expression of *Cdkn1c* resulted in the loss of iBAT. Mice inheriting a targeted deletion of *Cdkn1c* maternally (loss-of-function) die in the neonatal period [21]. *Cdkn1c*^-/+^ (KO^MAT^) embryos examined a day prior to neonatal demise, at E18.5, possessed poorly discernable iBAT depots lacking the characteristic butterfly shape normally observed at this stage of development (**Fig 6A**). H&E staining of the isolated KO^MAT^ depots revealed a disorganised morphology with large areas of lipid (**Fig 6B**). QPCR analysis confirmed considerably reduced expression of *Cdkn1c*. KO^MAT^ expressed relatively normal levels of *Rb1*, *PPARγ* and *C/EBPα* (**Fig 6C**). *C/EBPβ* was expressed at 50% the wild type level reciprocal to the increased expression observed in response to elevated *Cdkn1c*. Pan-adipocyte markers *fatty acid binding protein 4* (*Fabp4*) and *perilipin 1* (*Plin1*) were also markedly reduced. *Prdm16* was expressed at wild type levels while expression of *peroxisome proliferator-activated receptor gamma*, *coactivator 1 alpha* (*Ppargc1a*), which encodes a transcriptional coactivator that is involved in the activation of brown fat cells, was markedly elevated indicating that the initiation of brown adipocyte commitment was not prevented by loss of *Cdkn1c*. Nonetheless, there was a marked reduction in expression of downstream genes required for brown adipose development and function including the cAMP-inducible gene, *Ucp1*, and the cAMP insensitive genes *Cidea* and *Elovl3* (**Fig 6C**). Expression of the nuclear mitochondrial marker *Cycs* and the mitochondrion-encoded *Cox2* were also diminished, by 25–30% (**Fig 6C**). Mitochondrial DNA content was 40% less than the wild type level (**Fig 6D**). UCP1 and PRDM16 proteins were barely detectable in *Cdkn1c* KO^MAT^ iBAT (**Fig 6E and 6F**), all indicative of severely compromised BAT development. Loss of function of PRDM16 in iBAT early in life results in a switch from an iBAT identity towards a skeletal muscle identity [48]. Consistent with the loss of PRDM16, *Cdkn1c* KO^MAT^ iBAT expressed two-fold higher levels of the skeletal muscle-selective genes *myogenic factor 5* (*Myf5*) and *myogenic differentiation 1* (*Myod1*) (**Fig 6C**) further supported by western analysis for MYOD1 (**Fig 6G**). These data identified a requirement for *Cdkn1c* in the development of classic BAT.

### *Cdkn1c* induces a BAT-like gene program *ex-vivo*

**Fig 7:**
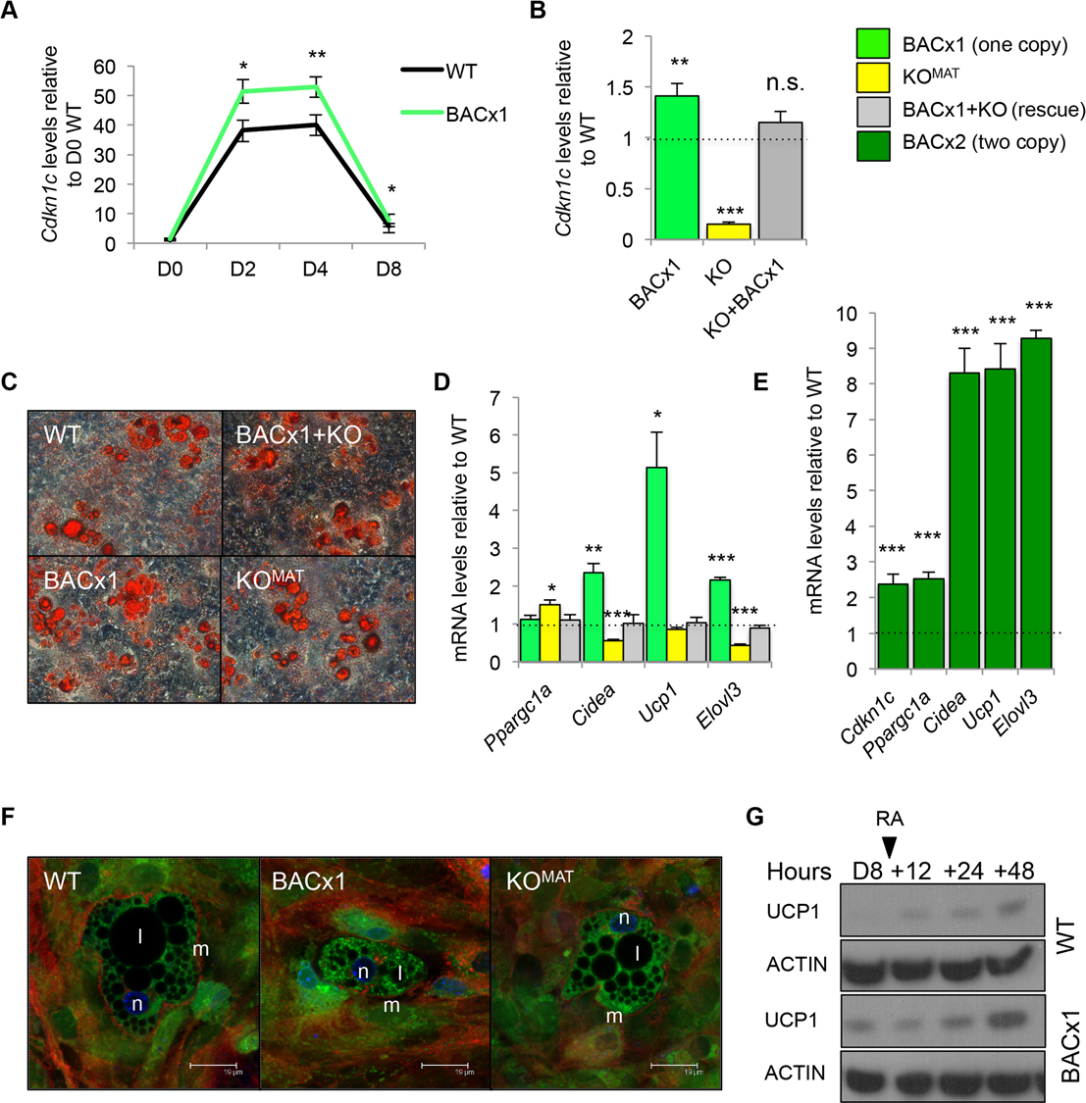
*Cdkn1c* induces a BAT-like gene program *ex-vivo*. (A) QPCR of analysis *Cdkn1c* mRNA levels in WT and BACx1 MEFs over 8 days of adipocyte induction relative to WT day (D) 0. (B) QPCR analysis of *Cdkn1c* expression in WT, BACx1, KO^MAT^ and BACx1+KO MEFs after 8 days of directed differentiation. (C) Oil Red O (ORO) staining of D8 adipocyte-differentiated WT, BACx1, KO^MAT^ and BACx1+KO MEFs. All genotypes produced lipid filled adipocytes. (D) QPCR analysis of BAT marker genes *Ppargc1a*, *Cidea*, *Ucp1*, and *Elovl3* in WT, BACx1, KO^MAT^ and BACx1+KO D8 adipocyte-differentiated MEFs. As *in vivo*, key markers of BAT fate and function were elevated. Critically BACx1+KO MEFs(*Cdkn1c* expressed at WT levels) expressed the BAT markers at WT levels confirming that induction was in response to the transgenic elevation of *Cdkn1c*. (E) QPCR analysis of *Cdkn1c* and BAT marker genes *Ppargc1a*, *Cidea*, *Ucp1*, and *Elovl3* in WT and BACx2 D8 adipocyte-differentiated MEFs illustrating further elevation of BAT markers driven by increased *Cdkn1c* dosage. (F) Confocal images of D8 adipocyte-differentiated WT, BACx1 and KO^MAT^ MEFs. Membranes stained with Cell mask Deep Red plasma (633nm; red), nuclei stained with Hoechst 366243 (450nm; blue) and mitochondria stained with Rhodamine-123 (540nm; green). Fields shown were visualised under fluorescence microscope at appropriate wavelengths. Mitochondria indicated by m, lipid by l and nucleus by n. Scale bar = 19 d. (G) Western analysis of UCP1 and β-ACTIN in D8 adipocyte-differentiated WT and BACx1 MEFs and after addition of 1 mM 9-*cis*-retinoic acid for 48 hours. UCP1 protein detectable by Western analysis in transgenic but not WT samples, an effect amplified by exposure to the positive regulator of *Ucp1* gene transcription, retinoic acid. For each QPCR analysis n = 4 genotypes per group taken from two independent litters; error bars represent ± s.e.m. * *P* <0.05; ** *P* <0.01; *** *P* <0.005.

To determine whether *Cdkn1c* could function intrinsically to boost brown adipogenesis, we performed an *ex-vivo* adipogenesis assay. Mouse embryonic fibroblasts (MEFs) are multipotent and have the potential to differentiate into brown adipocytes. MEFs were isolated from E12.5 BACx1 and WT fetuses and induced to differentiate using a standard adipogenic protocol [58]. The expression profile of *Cdkn1c* in both WT and BACx1 MEFs followed similar pattern of up regulation by day 2 and down regulation by day 8 of differentiation, with BACx1 MEFs expressing consistently higher levels of *Cdkn1c* at each time point (**Fig 7A**). Having confirmed elevated expression of *Cdkn1c* in the differentiating MEFs, a single copy of the *Cdkn1c* transgene was genetically combined with a maternally inherited targeted deletion of *Cdkn1c* (KO^MAT^) to generate MEFs of four genotypes: WT, BACx1, KO^MAT^ and KO+BACx1. After 8 days of adipocyte-directed differentiation *Cdkn1c* was expressed 1.4-fold the WT level in BACx1 MEFs and at barely detectable levels in KO^MAT^ MEFs (**Fig 7B**). KO+BACx1 MEFs, which carried both the transgene and the targeted allele, expressed *Cdkn1c* at WT levels (**Fig 7B**). All four genotypes differentiated into lipid-containing cells, as evidenced by Oil-Red O staining and mRNA levels for general adipogenic markers (**Fig 7C and S4 Fig**). As *in vivo*, key markers of BAT fate and function *Cidea*, *Ucp1* and *Elovl3* were elevated in BACx1 D8 MEFs (**Fig 7D**). Importantly, KO+BACx1 MEFs, in which *Cdkn1c* was expressed at WT levels, did not display altered expression of these markers (**Fig 7D**). After 8 days of adipocyte-directed differentiation BACx2 D8 MEFs displayed 2.4-fold elevated expression of *Cdkn1c* and further elevated expression of several BAT markers (**Fig 7E**) consistent with the dosage-related function of *Cdkn1c* in inducing a BAT-like gene program. Confocal imaging suggested more mitochondria in the BACx1 differentiated samples (**Fig 7F**), consistent with *in vivo* data (**Fig 4B**). UCP1 protein was detectable in BACx1 D8 MEFs but not WT MEFs, a difference further highlighted by exposure to the positive regulator of *Ucp1* gene transcription, retinoic acid [59, 60] (**Fig 7G**). Taken together, these data demonstrated that *Cdkn1c* can drive a BAT-like cell fate in adipocyte-differentiated fibroblast cells *ex-vivo*, and in a dosage-sensitive manner.

**Fig 8:**
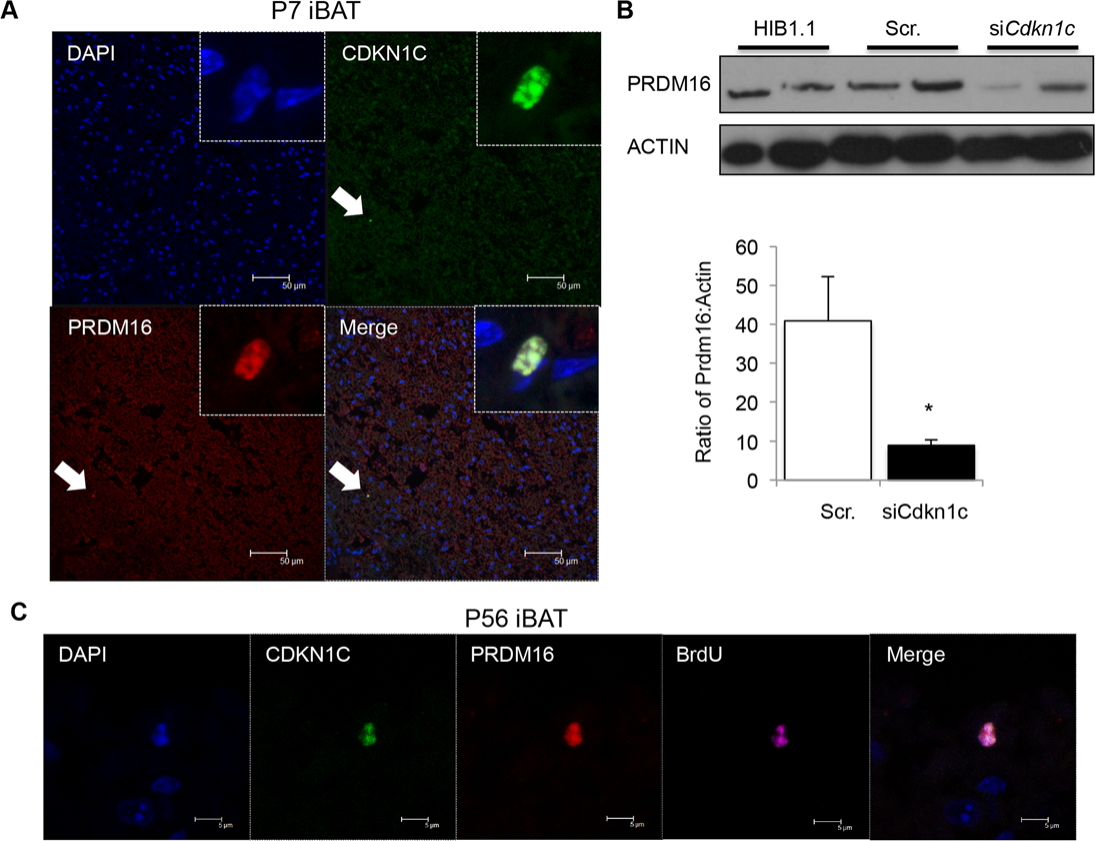
CDKN1C and PRDM16 co-localise to the nucleus of rare BrdU label-retaining cells in iBAT. (A) Confocal imaging of P7 iBAT co-stained for CDKN1C and PRDM16. DNA is stained with 4′,6-diamidino-2-phenylindole (DAPI, blue). (B) Western analysis of PRDM16 protein after siRNA-induced knock-down of *Cdkn1c* in the undifferentiated brown fat preadipocyte cell line HIB1.1. (C) Immunohistochemistry for CDKN1C (green), PRDM16 (red) and BRDU (purple) in WT iBAT 8 weeks after *in utero* pulsed exposure to BrdU. DNA (DAPI, blue).

There was a considerable loss of PRDM16 protein in *Cdkn1c* KO^MAT^ iBAT (**Fig 6F**) but *Prdm16* mRNA levels were relatively unaltered (**Fig 6C**) suggesting a function for *Cdkn1c* in the post transcriptional regulation of PRDM16. Consistent with this role, CDKN1C protein co-localised with the brown fat determinant, PRDM16, to the nucleus of rare cells present within P7 iBAT (**Fig 8A**). Acute loss of CDKN1C, driven by siRNA transfection of the brown preadipocyte cell line HIB-1B [61], resulted in a reduction of PRDM16 protein (**Fig 8B**). *Prdm16* and *Cdkn1c* are both known to be functionally important for adult haematopoietic stem cells [41, 62] and adult neural stem cells [63, 64]. 5-bromo-2-deoxyuridine (BrdU) label retention has been defined as a characteristic attributed to slow-cycling adult stem cells [65]. In two independent experiments, pregnant females were injected with BrdU (single dose at E16.5 or four doses of BrdU from E16.5). Within the adult iBAT from offspring of these pregnancies CDKN1C/PRDM16 double positive cells retained BrdU for six to eight weeks after embryonic labeling (**Fig 8C**). Taken together, all our data suggest that CDKN1C functions to support the post transcriptional accumulation of PRDM16 in a progenitor cell, and thus promotes the development of brown fat.

## Discussion

Here we provide *in vivo* evidence for key features of SRS in a novel mouse model of the minimal microduplicated region reported in some patients with this syndrome [66, 67] including low birth weight, head sparing, neonatal hypoglycaemia, smallness as adults and an extreme lack of body fat. Critically, we show that these phenotypes were due solely to the two-fold increased dosage of *Cdkn1c* consistent with the predicted expression levels in SRS patients. In our model, *Cdkn1c* was not ectopically expressed nor was *Cdkn1c* expressed at excessively high levels thus our findings are physiologically relevant. In addition to providing compelling evidence for a major role of elevated *CDKN1C* is SRS, we demonstrated *in vivo* and *ex-vivo* that *Cdkn1c* promotes the formation of brown adipose tissue, both the classic form exemplified by the iBAT depot and also the BIEGE form that emerges within WAT depots which persists into adulthood. Moreover, our data suggest that *Cdkn1c* functions to boost BAT, in part, by supporting protein accumulation of the brown fat determinant, *Prdm16*. This work has implications both for the diagnosis of SRS and the clinical management of SRS patients.

Microduplication mice were born low birth weight with a relative sparing of the head and neonatal hypoglycaemia. As adults the mice failed to catch-up in weight with their littermates and possessed substantially less white adipose tissue. We were able to exclude a role for two other genes present on the BAC (*Phlda2* and *Slc22a18)* in driving these phenotypes by using a reporter line in which expression of the BAC copy of *Cdkn1c* was replaced by lacZ [51]. While fetal growth restriction and low birth weight are relatively common complications of pregnancy that can have numerous origins, the more specific features of SRS support a major role for elevated *CDKN1C* expression in SRS. Currently the diagnosis of SRS is hampered by the complexity of alterations reported in different patients and the variable presentation of phenotypes. Moreover, some alterations may have an epigenetic origin not detectable by traditional DNA based approaches or not present in accessible tissues. The greater certainty that *CDKN1C* is a major contributor to SRS should lead to the development of better diagnostic tools and potentially the improved sub-classification of patients. It would now seem pertinent to examine BAT in SRS patients and, conversely, to assess individuals with a diagnosis of fetal growth restriction followed by extreme thinness for alterations in the expression of *CDKN1C*.

In addition to observing several defining features of SRS in our microduplication model, we identified *Cdkn1c* as a gene that functions *in vivo*, and in a dosage sensitive manner, to boost the amount of brown adipose tissue that develops early in life. Elevated *Cdkn1c* was associated with an increased amount of BEIGE adipose (non-classic BAT) located within the rpWAT depot in very young and in adult mice. Transgenic rpWAT depots had a marked appearance of BAT-like niches and expressed much higher levels of several BAT markers including *Elovl3* and *Cidea*, markers that are insensitive to cAMP. Both UCP1 and PRDM16 protein were readily detectable in BACx1 rpWAT depots in comparison to wild type depots in within litter comparisons. Elevated *Cdkn1c* also resulted in a larger iBAT depot relative to body weight in young mice and augmented the existing brown adipose gene program. The function of *Cdkn1c* in boosting the formation of BAT early in life would explain neonatal hypoglyceamia and the failure of our mice to lay down sufficient stores of white adipose tissue into adulthood manifesting as thinness.

While *Cdkn1c* was expressed from the BAC in a number of tissues including the pituitary, the hypothalamus and the pancreas [51] that may stimulate the browning of WAT, *Cdkn1c* was expression and imprinted within both rpWAT and iBAT depots. Importantly, elevated *Cdkn1c* enhanced the expression of brown adipose marker genes in adipogenically-differentiated MEFs. Normalising *Cdkn1c* by combining a single copy of the transgene with maternal inheritance of the targeted *Cdkn1c* allele in this same experiment resulted in wild type levels of both *Cdkn1c* and the BAT markers. This experiment demonstrated the intrinsic ability of *Cdkn1c* to drive a BAT-like gene program *ex-vivo*. Our findings that *Cdkn1c* plays a key role in promoting BAT development is novel and has important implications both for our understanding of BAT development.

Elevated *Cdkn1c* boosted the development of BAT while loss-of function of *Cdkn1c* resulted in abnormal morphology of the iBAT depot alongside a striking reduction in the expression of several brown adipose markers, and loss of UCP1 and PRDM16 protein. Classic iBAT derives from a common progenitor to skeletal muscle and a switch between these two lineages is thought to be controlled by *Prdm16* [48]. Consistent with loss-of-function of *Prdm16*, *Cdkn1c* KO iBAT expressed elevated levels of two muscle-specific genes. While PRDM16 protein was barely detectable, *Cdkn1c* KO iBAT expressed normal levels of *Prdm16* mRNA. Acute knock-down of CDKN1C in a brown fat cell line resulted in the loss of PRDM16 protein suggesting that *Cdkn1c* acts to regulate the post transcriptional accumulation of PRDM16. A precedent exists for *Cdkn1c* in regulating the post-transcriptional accumulation of several other transcription factors [30–36, 68, 69]. Moreover, co-expression of CDKN1C and PRDM16 in the nucleus of a rare, BrdU label-retaining cell in iBAT suggests that regulation takes place with an adult brown adipose progenitor cell. *Prdm16* and *Cdkn1c* are both already known to be functionally important for adult HSC [41, 62] and adult NSC [63, 64]. However, it remains controversial whether label retention is a definitive feature of stem cells and further work is required demonstrate that the PRDM16/CDKN1C double positive cells are indeed brown fat progenitors. What is clear is that both *Prdm16* and *Cdkn1c* are required for the proper determination of BAT cell fate, as evidenced by elevated expression of the myogenic markers *Myf5* and *MyoD* in response to loss-of-function of *Prdm16* [48] and *Cdkn1c* (**Fig 6**). Rather than participating in cell fate decisions, we propose that *Cdkn1c* modulates the accumulation of PRDM16 to promote “brownness”, acting downstream of cell fate choice.

Our mouse model recapitulated several defining features of SRS but there are potential limitations with this study. Firstly, the human and mouse CDKN1C predicted proteins share amino acid sequence conservation in the cyclin-dependent kinase inhibitory domain and in the QT domain, but the internal proline-rich and an acidic repeat domains found in the mouse sequence are replaced by a single PAPA repeat in the human sequence [27]. A key question that therefore arises is whether *CDKN1C* functions in humans to regulate brown adipogenesis? Although a low body mass index is consistent with more brown adipose tissue, we can find no report examining brown adipose tissue in SRS patients. However, recent data suggest that increased methylation at *CDKN1C* is associated with a higher BMI in a normal population [70] which holds promise. Secondly, while the transgenic model partially recapitulates the minimal microduplication observed in SRS, some key *Cdkn1c* enhancers located at a distance from the gene are absent from the mouse transgene [51]. We have examined the consequences of increased dosage in only a subset of tissues in which *Cdkn1c* is normally expressed, which excludes skeletal muscle and cartilage. However, this is likely to also be true for the SRS syndrome patients with smaller microduplications as the human *CDKN1C* enhancers are also located at a distance from the gene body [55].

Loss-of-function of *CDKN1C* in humans has been reported in cases of BWS. Excessive weight gain, which might be anticipated from a lack of BAT, is not a feature of BWS. BWS children can display neonatal hypoglycemia and one recent study reported early onset diabetes in a family with a mutation in *CDKN1C* [71], all of which could suggest a metabolic function for *CDKN1C* in humans. There are differences in the epigenetic regulation between humans and mice with some expression from the paternal allele in humans [55] which may attenuate the phenotype in BWS. Our findings may therefore have implications for several rare human imprinting disorders.

**Fig 9:**
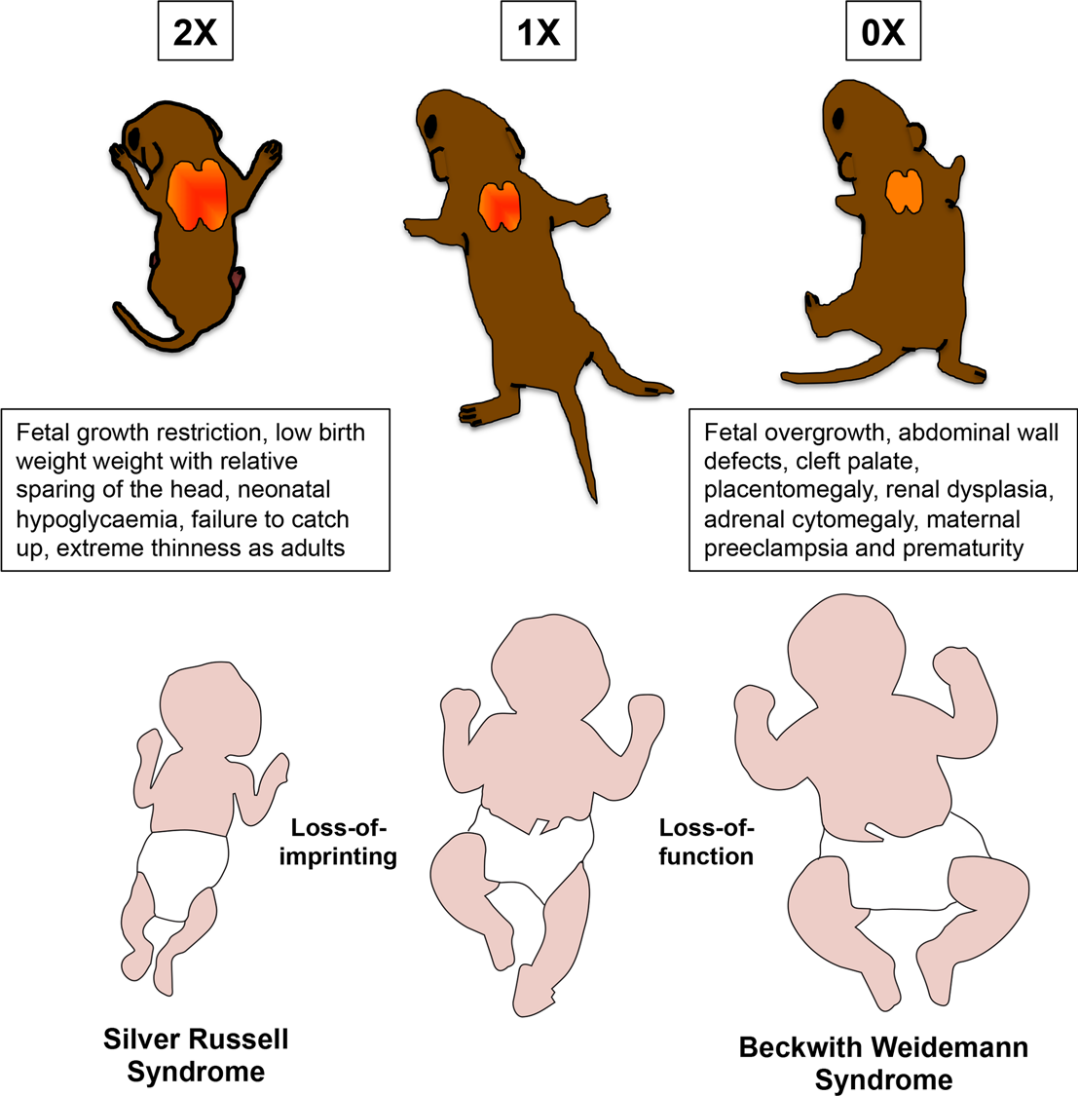
Two-fold *Cdkn1c* expression results in fetal growth restriction with characteristic features of Silver Russell Syndrome whereas loss-of function of *Cdkn1c* results in fetal overgrowth with characteristic features of Beckwith Weidemann Syndrome.

There is now sufficient evidence from animal models and human studies to indicate a key role for the imprinted *CDKN1C* gene in SRS, BWS and IMAGe syndrome (**Fig 9**). This knowledge will undoubtedly improve our understanding of these complex childhood growth disorders and their longer term implications. From an evolutionary perspective, our finding that *Cdkn1c* acts early in life to promote the formation of brown adipose tissue in mice is also intriguing. Thermogenesis is critical for the survival of young mammals before the development of subcutaneous fat and hair but comes at an energetic cost to the individual. *Cdkn1c* is both a BAT-promoting gene and one that negatively regulates embryonic growth [24, 25]. Our data predict that silencing of *Cdkn1c* by the paternal genome, which occurred after mammals diverged from marsupials [72], would result in larger offspring with the simultaneous reallocation of resources away from maintaining body temperature towards supporting the enhanced growth, providing a clear competitive advantage and lending support to the hypothesis that thermogenesis is an arena for genomic conflict in mammals [73].

In conclusion, this work provides genetic evidence from a physiological relevant animal mode that *Cdkn1c* functions to boosts the development of BAT in mice. This work fundamentally establishes that *Cdkn1c* gene dosage, rather than gene function *per se*, plays a key role in this process. We critically show that relatively small (< two-fold) changes in gene expression can have a dramatic consequence for development in mice with long lasting consequences. If these functions hold true in humans, this information will provide a step change in our understanding of the pathologies that occur in SRS and potentially other disorders including BWS and IMAGe syndrome, leading to improved diagnosis and the clinical management of patients.

## Materials and Methods

### Animals and husbandry

All animal studies and breeding was approved by the University of Cardiff ethical committee and performed under a UK Home Office project license (RMJ). Mice were housed on a 12 hour light–dark cycle with lights coming on at 06.00 hours with a temperature range of 21°C +/- 2 with free access to tap water and standard chow. BAC transgenic lines *Cdkn1c*^*BACx1*^, *Cdkn1c*^*BACx2*^ and *Cdkn1c* ^*BAC-lacZ*^, were bred onto a C57BL/6J (BL6) background for >12 generations and genotyped as described [51]. The *Cdkn1c*^*tm1Sje*^ allele [21] for historical reasons was maintained on the 129S2/SvHsd (129) background. Cdkn1c-RFLP mice were generated by crossing a *M*. *m*. *spretus* male with a BL6 female and selecting for the *Cdkn1c AvaI* RFLP for >8 generations. Basal body temperatures of group-housed, experimentally naive female transgenic mice were monitored with a rectal probe (IN005A, Vet Tech solution). Surface temperature of P2 pups was recorded using a thermal imaging camera (Optris P1200). Glucose concentrations in whole blood were determined in neonatal pups in the fed state with the HemoCue system.

### Histological analyses, in situ hybridisation and immunohistochemistry

*β-galactosidase* (lacZ) staining, H&E staining and *in situ* hybridisation were performed as previously described [51]. CDKN1C immunohistochemistry: 10 μm sections were prepared from E16.5 fetuses fixed overnight in 4% PFA at 4°C and paraffin embedded. Slides were dewaxed in xylene and rehydrated through graded ethanols, submerged in 1X Citrate Buffer (DAKO) and heated in a pressure cooker for 20 minutes. Slides were cooled and blocked for 20 minutes in Peroxidase Block (Envsion), then 30 minutes in 10% normal rabbit serum and 1% BSA in PBS, and then incubated in primary antibody (Santa Cruz P57 M-20; SC-1039) overnight at room temperature diluted 1:50 in 10% rabbit serum and 1% BSA in PBS, washed in PBS, incubated with 1:200 dilution of HRP-conjugated rabbit anti-goat IgG secondary (DAKO) for 1 hour at room temperature, washed in PBS 3 x 5 minutes at room temperature and visualized with DAB (DAKO). Slides were counterstained in Mayers Haematoxylin, dehydrated, cleared and mounted in DPX mounting medium. For electron microscopy, rpWATs from P7 mice were fixed overnight in 2% PFA/2% gluteraldehyde in 0.1M Sorensons PB, post fixed in 1% osmium tetraoxide for 2 hours and stained in uranyl acetate overnight at 4°C. After sequential dehydration, samples were embedded in pure araldite and ultra-thin sections were visualised under Philips TEM 208 transmission electron microscope (Phillips). Cryosections were incubated with primary antibodies (1:100 dilution) for 3 hours at room temperature, washed in PBS before incubation with fluorescent secondary antibodies (1:1000 dilution) for one hour at 4°C followed by 4’,6-diamidino-2-phenylindole (DAPI) staining. Slides were mounted using Fluoromount aqueous media (Sigma) and imaged using Leica TCS SP2 AOBS laser confocal microscope, and Leica Confocal software. Samples were scanned with appropriate excitation and emission settings (**S2 Table**). To identify label-retaining cells in iBAT, we performed two BrdU pulse chase experiments. WT pregnant mice were intraperitoneally administered injections of BrdU at 80 mg/kg/time (Sigma, USA) either once at E16.5 or twice daily from E16.5 for two days. Offspring born from these pregnancies were euthanised 6–8 weeks after the last BrdU injection. iBAT was harvested and cryosections were incubated with the primary antibodies to CDKN1C, PRDM16, BRDU and fluorescent secondary antibodies as described above. Samples were scanned with appropriate excitation and emission settings (**S3 Table**).

### DNA, RNA and protein analysis

Genomic DNA was bisulphite treated using an EZ DNA Methylation Kit (Zymo Research). Sodium modification treatments were carried out in duplicate for each DNA sample and at least three independent amplification experiments were performed for each individual examined. The region spanning the *Cdkn1c* was amplified by PCR using primers 5’-tgggtgtagagggtggatttagtta-3’s and 5’- cccacaaaaaccctaccccc-3’ and hemi-nested primer 5’- gtattgttaggattaggatttagttggtagtagtag. The PCR products were cloned into pGEM-T (Promega, Madison, WI, USA) and an average of 20 clones per sample were sequenced using M13 reverse primer and an automated ABI Prism 3130xl Genetic Analyzer (Applied Biosystems, Foster city, CA, USA) as previously described [74]. Quantitative RT-PCR was performed in duplicate on four independent samples obtained from two litters as described [75]. Mitochondrial DNA was quantitated by comparing the nuclear mitochondrial marker *cytochrome c*, *somatic* (*Cycs*) with the mitochondrion-encoded *cytochrome c oxidase subunit II* (*Cox2*) by quantitative PCR. Primers are given in **S1 Table**. RFLP analysis was performed on cDNA prepared from iBAT and rpWAT obtained from crossing a BL6 female with a Cdkn1c-RFLP male. Western blot analysis: total proteins (30 μg) were resolved by SDS-PAGE, transferred to PVDF (Millipore Corp., Bedford, MA), blocked in TBS-T (10 mM Tris, 150 mM NaCl, 0.05% Tween 20, 5% skimmed milk), incubated with primary antibodies (Sigma SAB4500071 CDKN1C; Sigma SAB1300006 PRDM16; Abcam ab10983 UCP1; R&D sytems MAB5966 MYOD; Sigma A5316 β-ACTIN) and visualised using secondary horseradish peroxidase-linked antibodies (Invitrogen) and ECL.

### Cell culture

For differentiation experiments, MEFs isolated from E12.5 embryos and cultured in DMEM/F12 (Invitrogen), 10% fetal bovine serum (Invitrogen), 2 mM glutamine (Sigma) and 50 μg/ml penicillin/streptomycin (Sigma) for two passages were used. Differentiation of two-day-post confluent MEFs (D0) was performed by incubation with 170 nM insulin (Sigma), 250 nM dexamethasone (Sigma), 2.5 nM rosiglitazone (Axxora ALX-350-125-M025) and 0.5 mM isobutylmethylxanthine (IBMX) (Sigma) for 2 days and medium containing only 170 nM insulin and 2.5 nM rosiglitazone for 6 additional days, changing the medium every 48 hours. For ORO, cells were fixed for 20 minutes in paraformaldehyde vapour and stained for 15 minutes with Oil Red O solution (0.6% (w/v) in isopropanol:water 60:40), washed and photographed. For UCP1 western blots, cells differentiated for 8 days were harvested, or treated with vehicle (dimethyl sulfoxide) or 9-cis-retinoic acid (1 μM in dimethyl sulfoxide) over 48 hours with protein extraction at intervals. For confocal microscopy, MEFs underwent differentiation in 5 cm glass bottom plates (Mat Tek). After 8 days of differentiation, cells were stained with 5 μg/ml Hoechst 33342 (Invitrogen) and Rhodimine-123 (Sigma Aldrich) for 30 minutes at 37°C. Dyes were removed, and cells were washed for 5 minutes in media. Further staining with 7.5 μg/ ml HCS CellMask Red (Invitrogen) for 10 minutes was performed followed by three washes in ddH_2_0. Samples were imaged using Leica TCS SP2 AOBS laser confocal microscope and Leica Confocal software. HIB-1B cells were maintained in DMEM/F12 (Invitrogen) supplemented with 10% fetal bovine serum (Invitrogen), 2 mM glutamine (Sigma) and 50μg/ml penicillin/streptomycin (Sigma). The siRNA sequence for *Cdkn1c*-depletion was *p57* siRNA (m) (Santa Cruz Biotechnology sc-37621). Control siRNA-A (Santa Cruz Biotechnology sc-37007) was used as the scrambled sequence. Lipid complexes were prepared and reverse transfected according to manufacturer instructions (INTERFERin, Polyplus) in 12-well plates with 10 pmole of the siRNAs complexed with 2 μl of INTERFERin in OPTIMEM with a repeat transfection performed at 24 hours. Cells were harvested 48 hours after transfection and analysed by western blotting. Experiments were performed in three separate occasions in duplicate (ECL) or triplicate (fluorescent) wells.

### Statistical analyses

Statistical significance (Probability values) was determined using the Student’s t-Test (two tailed distribution and two sample unequal variance). For qPCR analysis, Mann-Whitney test was performed on ∆Ct values between groups.

## Supporting Information

S1 Fig Assessment of fetal weights and survival on C57BL/6J strain background. (A) E16.5 weight data for BACx1, BACx2 and BAClaz (B) E18.5 weight data for BACx1, BACx2 and BAClaz (C) CHI-SQUARED χ^2^ test. Null = no difference in n; Alternative = difference in n; Critical Value 3.841; P = 0.05; Degrees of Freedom = 1
(TIF)

S2 Fig **Magnetic resonance images showing fat deposition in non-transgenic (A) and BACx1 transgenic (B) adult male littermates on a mixed 129/BL6 genetic background.** Two multislice image sets were obtained without and with chemical shift selective fat suppression to generate fat only images (right). Transgenic males had visibly less subcutaneous and visceral fat (white signal).
(TIF)

S3 Fig Assessment of BAC-lacZ reporter adipose depots. (A) H&E staining of WT and BAC-lacZ P7 rpWAT. (B) QPCR of BAT markers in WT and BAC-lacZ P7 rpWAT. (C) H&E staining of WT and BAC-lacZ P7 iBAT. (D) QPCR of BAT markers in WT and BAC-lacZ P7 iBAT. WT and reporter line are morphologically indistinguishable and exhibit wild type levels of *Cdkn1c*, *Ucp1*, *Elovl3* and *Prdm16* genetically attributing the rpWAT and iBAT alterations in BACx1 and BACx2 pups to elevated expression of *Cdkn1c*.
(TIF)

S4 Fig QPCR analysis of markers of adipocyte differentiation for adipocyte-differentiated MEFs. *Rb1*, *PPARγ*, *C/EBPα* and *C/EBPβ* in WT, BACx1 and KO^MAT^ adipocyte-differentiated MEFs (same samples as shown in **Fig 7D**).
(TIF)

S1 Table QPCR primers. (DOCX)

S2 Table Immunofluorescence primary and secondary antibodies used to generate data shown in Fig 8A. (DOCX)

S3 Table Immunofluorescence primary and secondary antibodies used to generate data shown in Fig 8C. (DOCX)
