# Supplementary figures and images for: Cdkn1c Boosts the Development of Brown Adipose Tissue in a Murine Model of Silver Russell Syndrome
Source: PLoS Genet. 2016 Mar 10;12(3):e1005916. doi: 10.1371/journal.pgen.1005916 (PMC4786089; doi:10.1371/journal.pgen.1005916)

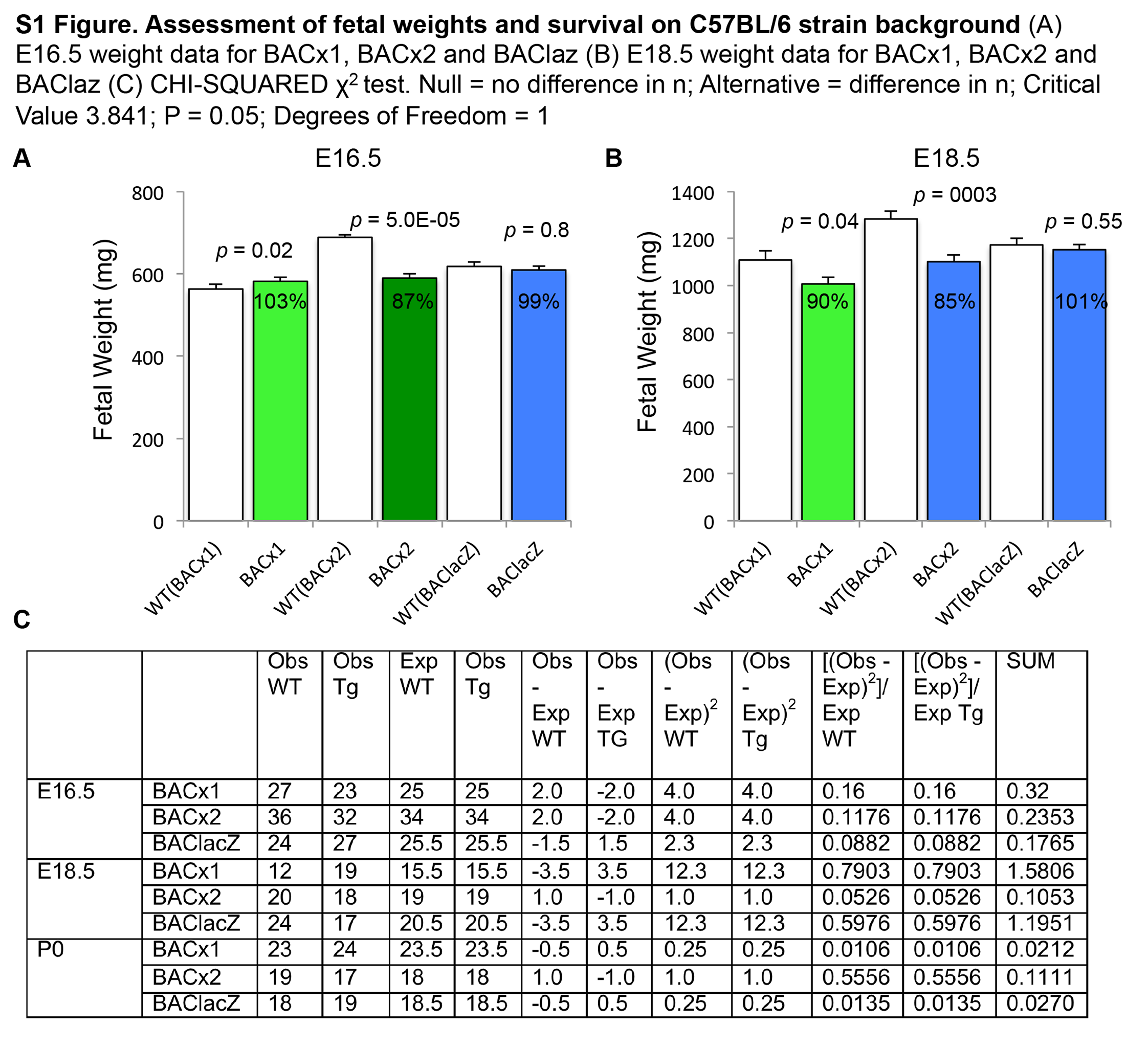

Supplement: S1 Fig — (A) E16.5 weight data for BACx1, BACx2 and BAClaz (B) E18.5 weight data for BACx1, BACx2 and BAClaz (C) CHI-SQUARED χ2 test. Null = no difference in n; Alternative = difference in n; Critical Value 3.841; P = 0.05; Degrees of Freedom = 1 (TIF) [file pgen.1005916.s001.tif]

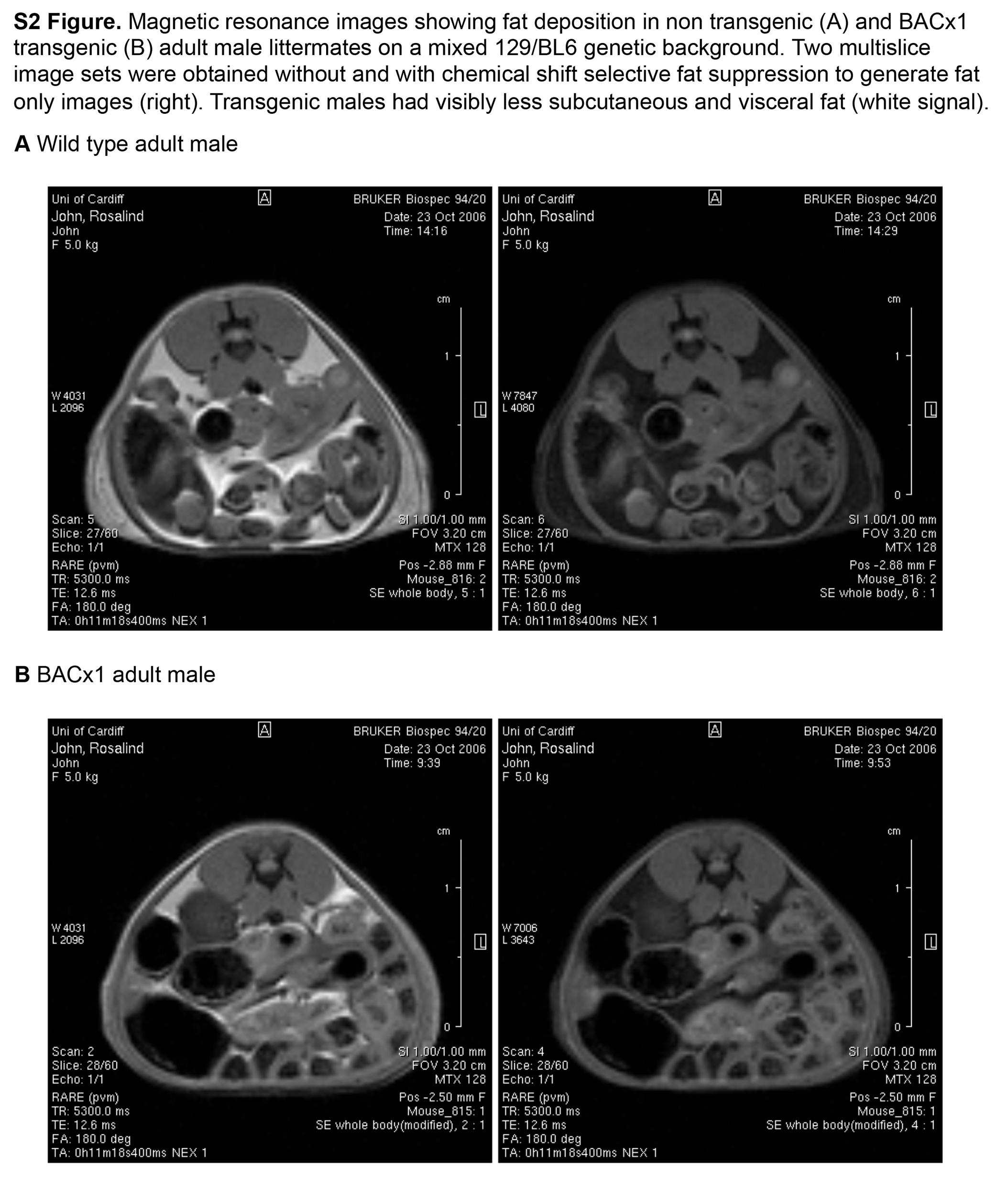

Supplement: S2 Fig — Magnetic resonance images showing fat deposition in non-transgenic (A) and BACx1 transgenic (B) adult male littermates on a mixed 129/BL6 genetic background. Two multislice image sets were obtained without and with chemical shift selective fat suppression to generate fat only images (right). Transgenic males had visibly less subcutaneous and visceral fat (white signal). (TIF) [file pgen.1005916.s002.tif]

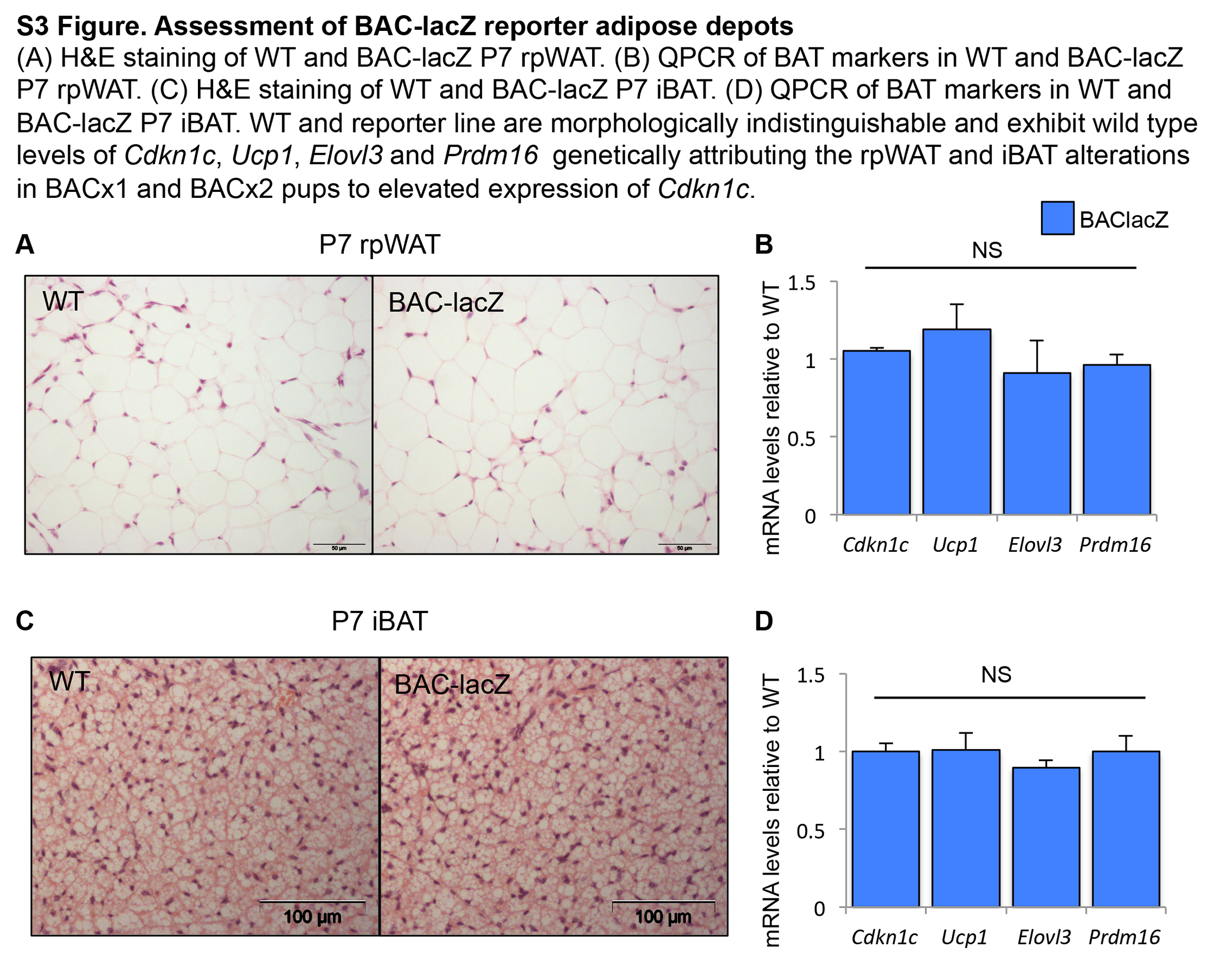

Supplement: S3 Fig — (A) H&E staining of WT and BAC-lacZ P7 rpWAT. (B) QPCR of BAT markers in WT and BAC-lacZ P7 rpWAT. (C) H&E staining of WT and BAC-lacZ P7 iBAT. (D) QPCR of BAT markers in WT and BAC-lacZ P7 iBAT. WT and reporter line are morphologically indistinguishable and exhibit wild type levels of Cdkn1c, Ucp1, Elovl3 and Prdm16 genetically attributing the rpWAT and iBAT alterations in BACx1 and BACx2 pups to elevated expression of Cdkn1c. (TIF) [file pgen.1005916.s003.tif]

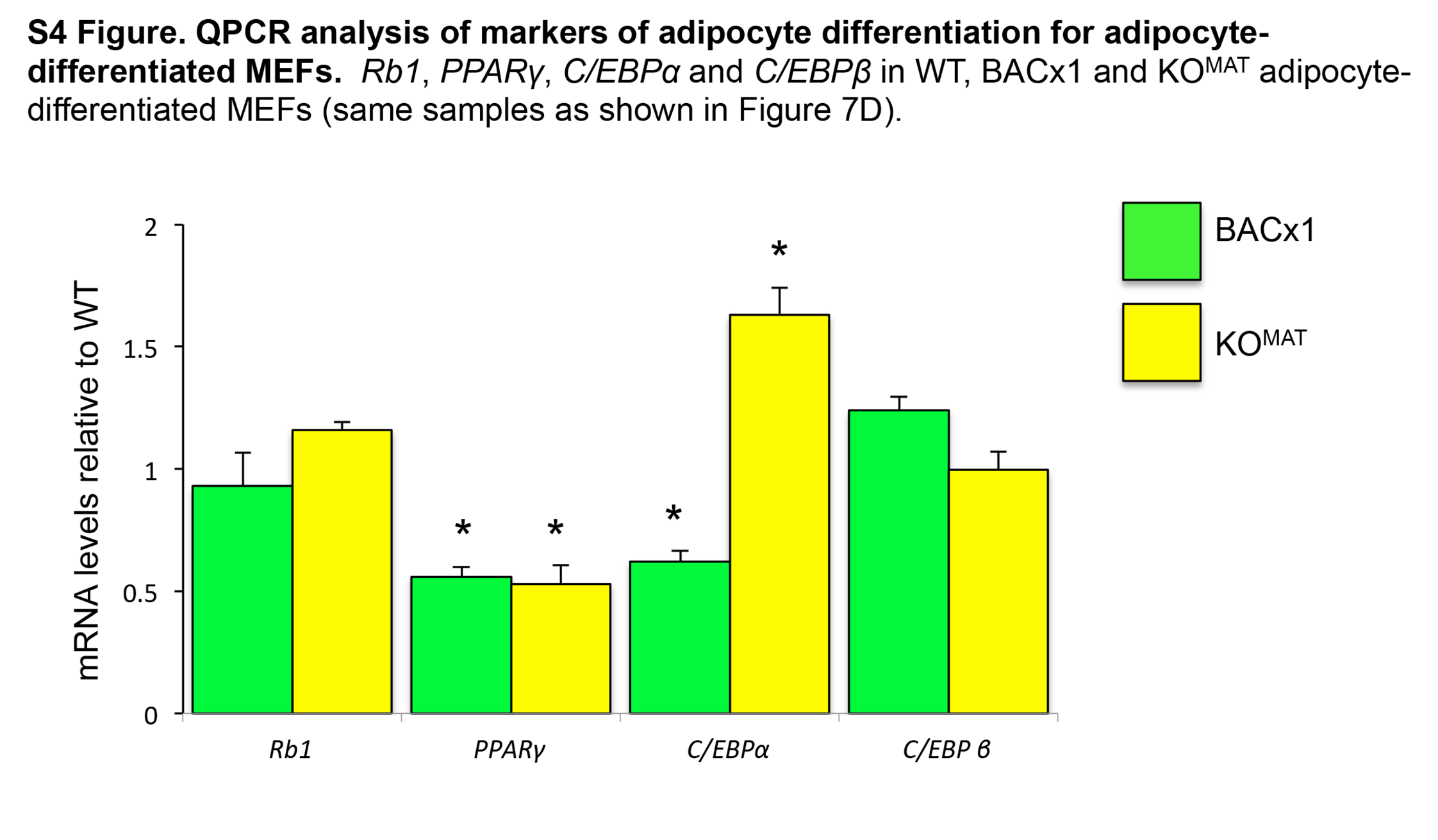

Supplement: S4 Fig — Rb1, PPARγ, C/EBPα and C/EBPβ in WT, BACx1 and KOMAT adipocyte-differentiated MEFs (same samples as shown in Fig 7D). (TIF) [file pgen.1005916.s004.tif]
