# Supplementary material for: Cdkn1c Boosts the Development of Brown Adipose Tissue in a Murine Model of Silver Russell Syndrome
Source: PLoS Genet. 2016 Mar 10;12(3):e1005916. doi: 10.1371/journal.pgen.1005916 (PMC4786089; doi:10.1371/journal.pgen.1005916)
Supplement: S1 Table — (DOCX) [file pgen.1005916.s005.docx]

**S1 Table: QPCR primers**

| Gene | 5’ primer | 3’ primer |
| --- | --- | --- |
| *Cdkn1c* | agagaactgcgcaggagaac | tctggccgttagcctctaaa |
| *Ucp1* | ggcaaaaacagaaggattgc | taagccggctgagatcttgt |
| *Cidea* | tggaaaagggacagaaatgg | tctcgtacatcgtggctttg |
| *pRb* | atttgtccttcccgtggatt | gaggacaagcaggttcaagg |
| *PPARγ* | ccctggcaaagcatttgtat | gaaactggcacccttgaaaa |
| *Ppargc1a (PGC-1α)* | tcatcacctaccgttacacctg | caagcttctctgagcttccttc |
| *C/EBPα* | cgctggtgatcaaacaagag | cgatctggaactgcaagtgg |
| *C/EBPβ* | caagctgagcgacgagtaca | agctgctccaccttcttctg |
| *Fsp-27* | gacccaacagctggtgtctaa | attgtgccatcttcctccag |
| *Plin1 (perilipin 1)* | cactctctggccatgtggat | agaggctgccaggttgtg |
| *Elovl3* | ctgttgctcatcgttgttgg | atctgactacggcgtcatcc |
| *Fabp4 (aP2)* | tcacctggaagacagctcct | aatccccatttacgctgatg |
| *Cycs (Cyt C)* | tccatcagggtatcctctcc | ggaggcaaccataagactgg |
| *Cox 2* | aattgctctcccctctctacg | gtagcttcagtatcattggtgc |
| *PRDM16* | aaaccatgacggagaagctg | aggttggaggagatgctgaa |
| *PRDM16* | cagcacggtgaagccattc | gcgtgcatccgcttgtg |
| *Actb (β-Actin)* | cctgtatgcctctggtcgta | ccatctcctgctcgaagtct |
| *GAPDH* | cacagtcaaggccgagaatg | tctcgtggttcacacccatc |
| *Myf5* | cagccccacctccaactg | gggaccagacagggctgtta |
| *MyoD* | cgccactccgggacatag | gaagtcgtctgctgtctcaaagg |
