# Supplementary material for: Cdkn1c Boosts the Development of Brown Adipose Tissue in a Murine Model of Silver Russell Syndrome
Source: PLoS Genet. 2016 Mar 10;12(3):e1005916. doi: 10.1371/journal.pgen.1005916 (PMC4786089; doi:10.1371/journal.pgen.1005916)
Supplement: S3 Table — (DOCX) [file pgen.1005916.s007.docx]

**S3 Table: Immunofluorescence primary and secondary antibodies used to generate data shown in Figure 8C.**

| Primary | Secondary | Ex max | Em max | Pseudo colour |
| --- | --- | --- | --- | --- |
| DAPI |  | 358 | 461 | blue |
| goat anti-Cdkn1c (Santa Cruz sc-1039) | Alexa Fluor 546 donkey anti-goat (red) | 566 | 573 | green |
| mouse anti-BrdU  (BD Biosciences 347580) | Alexa Fluor 488  donkey anti-mouse (green) | 495 | 519 | purple |
| rabbit anti-Prdm16 (Sigma SAB1300006) | Alexa Fluor 633 goat anti-rabbit (far red, A-21070) | 632 | 647 | red |
